# MucoRice-CTB line 19A, a new marker-free transgenic rice-based cholera vaccine produced in an LED-based hydroponic system

**DOI:** 10.3389/fpls.2024.1342662

**Published:** 2024-03-15

**Authors:** Yoshikazu Yuki, Shiho Kurokawa, Kotomi Sugiura, Koji Kashima, Shinichi Maruyama, Tomoyuki Yamanoue, Ayaka Honma, Mio Mejima, Natsumi Takeyama, Masaharu Kuroda, Hiroko Kozuka-Hata, Masaaki Oyama, Takehiro Masumura, Rika Nakahashi-Ouchida, Kohtaro Fujihashi, Takashi Hiraizumi, Eiji Goto, Hiroshi Kiyono

**Affiliations:** ^1^ Division of Mucosal Immunology, IMSUT Distinguished Professor Unit, The Institute of Medical Science, The University of Tokyo, Tokyo, Japan; ^2^ R&D department, HanaVax Inc., Chiba, Japan; ^3^ Department of Human Mucosal Vaccinology, Chiba University Hospital, Chiba, Japan; ^4^ Technical Research Institute, Asahi Kogyosha Co., Ltd., Tokyo, Japan; ^5^ Research Department, Nisseiken Co., Ltd., Tokyo, Japan; ^6^ Division of Genome Editing Research, National Agriculture and Food Research Organization, Tsukuba, Japan; ^7^ Medical Proteomics Laboratory, The Institute of Medical Science, The University of Tokyo, Tokyo, Japan; ^8^ Laboratory of Genetic Engineering, Graduate School of Agriculture, Kyoto Prefectural University, Kyoto, Japan; ^9^ Future Mucosal Vaccine Research and Development Synergy Institute, Chiba University, Chiba, Japan; ^10^ Department of Pediatric Dentistry, The University of Alabama at Birmingham, Birmingham, AL, United States; ^11^ Graduate School of Horticulture, Chiba University, Chiba, Japan; ^12^ Mucosal Immunology and Allergy Therapeutics, Institute for Global Prominent Research, Research Institute of Disaster Medicine, Chiba University Future Medicine Education and Research Organization, Chiba University, Chiba, Japan; ^13^ CU-UCSD Center for Mucosal Immunology, Allergy, and Vaccine (cMAV), Division of Gastroenterology, Department of Medicine, University of California, San Diego, San Diego, CA, United States

**Keywords:** MucoRice, rice-based vaccine, cholera, oral vaccine, vaccine development

## Abstract

We previously established the selection-marker-free rice-based oral cholera vaccine (MucoRice-CTB) line 51A for human use by *Agrobacterium*-mediated co-transformation and conducted a double-blind, randomized, placebo-controlled phase I trial in Japan and the United States. Although MucoRice-CTB 51A was acceptably safe and well tolerated by healthy Japanese and U.S. subjects and induced CTB-specific antibodies neutralizing cholera toxin secreted by *Vibrio cholerae*, we were limited to a 6-g cohort in the U.S. trial because of insufficient production of MucoRice-CTB. Since MucoRice-CTB 51A did not grow in sunlight, we re-examined the previously established marker-free lines and selected MucoRice-CTB line 19A. Southern blot analysis of line 19A showed a single copy of the *CTB* gene. We resequenced the whole genome and detected the transgene in an intergenic region in chromosome 1. After establishing a master seed bank of MucoRice-CTB line 19A, we established a hydroponic production facility with LED lighting to reduce electricity consumption and to increase production capacity for clinical trials. Shotgun MS/MS proteomics analysis of MucoRice-CTB 19A showed low levels of α-amylase/trypsin inhibitor-like proteins (major rice allergens), which was consistent with the data for line 51A. We also demonstrated that MucoRice-CTB 19A had high oral immunogenicity and induced protective immunity against cholera toxin challenge in mice. These results indicate that MucoRice-CTB 19A is a suitable oral cholera vaccine candidate for Phase I and II clinical trials in humans, including a *V. cholerae* challenge study.

## Introduction

Many conventional vaccines against infectious diseases have to be administered by injection and to be stored in refrigerators because they are unstable at higher temperatures. Although next-generation vaccine technologies based on mRNA and the adenovirus vector system have been developed in response to the global SARS-Cov-2 pandemic, these are systemic vaccines and cannot be used as mucosal vaccines and need to be stored in a freezer or refrigerator (Carvalho et al., 2021). Vaccines that are stable at room temperature and can be administered without syringes and needles are eagerly awaited worldwide (Varmus et al., 2003). In two decades, many public health insurance organizations, including the WHO, have proposed and are promoting the development of such vaccines that can be administered orally (Kartoglu and Ames, 2022). However, technical difficulties have hampered the development of inexpensive oral vaccines that are, stable at room temperature.

Diarrheal diseases including cholera are an important global issue (Ali et al., 2015). Oral cholera vaccines containing inactivated *Vibrio cholerae* only (Shanchol from Sanofi Pasteur and Euvichol from Eubiologics) (Qadri et al., 2016; Odevall et al., 2018) or inactivated *V. cholerae* plus recombinant cholera toxin (CT) B chain (CTB) against cholera and traveler’s diarrhea (Dukoral from Sanofi Pasteur) (Clemens et al., 1986, Clemens et al., 1988) have been licensed, but they need to be kept in refrigerators. MucoRice-CTB is an oral vaccine candidate for cholera and traveler’s diarrhea that expresses the CTB antigen in the endosperm cells of rice seeds and is stable at room temperature; this technology has enabled the development of cold chain–free (room temperature–stable) oral vaccines against enteric infections (Nochi et al., 2007). This vaccine is effective in suppressing diarrhea in a CT challenge test in orally immunized mice (Nochi et al., 2007; Yuki et al., 2009) and in a cholera intestinal loop test (Tokuhara et al., 2010). The vaccine was also effective against diarrhea in a heat-labile enterotoxin (LT) challenge test and enteric loop assay in LT-producing pathogenic *Escherichia coli*, in which LT is homologous to cholera toxin (Tokuhara et al., 2010; Takeyama et al., 2015). Cholera and traveler’s diarrhea, which affects an estimated 10 million people worldwide (Girard et al., 2006), are covered by this vaccine. Importantly, CTB administered as an injectable vaccine is not effective in suppressing diarrhea despite the induction of antigen-specific serum IgG antibodies; Oral immunization with MucoRice-CTB provided effective SIgA-mediated protection against CT- or LT-induced diarrhea, but the protection was impaired in polymeric Ig receptor–deficient mice lacking SIgA (Tokuhara et al., 2010).

The prototype selection marker–free MucoRice-CTB 51A has been used as a vaccine in Phase I studies in Japan and the U.S.; they confirmed its safety and tolerability up to 6 g in oral administration, as well as induction of CTB-specific antibodies that neutralize cholera toxin in the serum, but the U.S. trial was limited to 6 g because of vaccine shortage (Yuki et al., 2021, Yuki et al., 2022).

In the present study, we reviewed marker-free lines established in the past (Mejima et al., 2015) and selected a new vaccine candidate, MucoRice-CTB line 19A, which can grow under sunlight and LED and can be used for scale-up, since MucoRice-CTB 51A line does not grow under sunlight or LED light. In addition, we characterized the genetic, proteomic, physiological, and immunological properties of MucoRice-CTB line 19A as a new marker-free cholera vaccine produced in a closed-type hydroponic system with LED lighting. A Cartagena Protocol on Biosafety–compliant hydroponic production facility equipped with LED lighting for the MucoRice-CTB line 19A for preclinical studies was installed at the Faculty of Horticulture, Chiba University, in order to reduce the power consumption of the metal halide lamp by one-tenth and increase the production capacity.

## Materials and methods

### Comparison of four marker–free MucoRice-CTB lines

We previously developed a uniform rice-derived oral cholera vaccine (MucoRice-CTB) using an overexpression system of CTB (N4Q), a modified cholera toxin B subunit, and RNAi to inhibit the production of key rice endogenous storage proteins such as a prolamin and glutelin (Yuki et al., 2013). To establish MucoRice-CTB for human use, we developed MucoRice-CTB without the hygromycin phosphotransferase (HPT) selection marker by co-transforming two *Agrobacterium tumefaciens*, each with different T-DNA. (**Supplementary Figure 1**) and had produced six selection maker–free MucoRice-CTB lines. Of the 6 lines advanced to the T4 generation (Mejima et al., 2015), we used MucoRice-CTB lines 19A, 9B, 43B, and 50A in this paper (**Supplementary Table 1**). The T6 generation of MucoRice-CTB line 19A was produced as a master seed bank in the MucoRice-CTB production facility at the Institute of Medical Science, University of Tokyo, on September 28, 2016.

### PCR analysis

Leaf genomic DNA was isolated with a Nucleon PhytoPure kit (GE Healthcare, WI, USA). PCR was performed by using the EmeraldAmp MAX PCR Master Mix (PR320A, Takara, Tokyo, Japan)) on a Veriti Thermal Cycler (Thermo Fisher Scientific) as follows: 35 cycles of 30 s at 94°C, 30 s at 60°C, and 1 min at 72°C. The reaction products were separated by electrophoresis on a 1.5% (w/v) agarose gel. To determine full transgene sequences, PCR was performed with KOD FX polymerase (KFX-101, Toyobo, Osaka, Japan) on the same thermal cycler as follows: 2 min at 94°C and 35 cycles of 10 s at 98°C, 5 min at 68°C. The reaction products were separated by electrophoresis on a 0.7% (w/v) agarose gel. All primers used are listed in **Supplementary Table 2**. The pZAAMP-CTB-10Li45GB3A and pZH2B plasmids were used as positive controls, and WT genomic DNA was used as a negative control.

### Quantitative real-time PCR

Seeds of non-transgenic *Oryza sativa* L cv. Nipponbare (WT) and MucoRice-CTB lines were harvested 14 days after flowering. Total RNA was extracted with an RNeasy Plant Mini Kit (Qiagen, Hilden, Germany) and treated with DNase (Takara Bio Inc.). Total RNA (0.5 µg) was used to synthesize cDNA with a Prime Script Reagent Kit with gDNA Eraser (Takara Bio Inc.). cDNA (20 µl) was diluted 50 times with distilled water and used as a template. PCR was performed in 20 µL; the Fast SYBR Green Master Mix (Applied Biosystems) and a StepOne Plus real-time PCR system (Applied Biosystems) were used. The primer sets are shown in **Supplementary Table 2**. Triplicate reactions were performed as follows: 20 s at 95°C; 40 cycles of 3 s at 95°C, 30 s at 60°C, 15 s at 95°C; and 1 min at 60°C. Transcript levels were normalized to that of 17S RNA. The specificity was assessed by analyzing the product melting curves.

### Southern blot analysis

Leaf genomic DNA was extracted from young leaves (2 g) as above. Standard procedures (Sambrook et al., 1989) were used. Genomic DNA (~20 µg) was digested with EcoRI or SacI, followed by fractionation on a 0.7% (w/v) agarose gel and blotting onto a Hybond N+ nylon membrane (GE Healthcare). The *CTB* DNA probe was amplified from pZAAMP-CTB-10Li45GB3A by PCR with the CTB-F and -R primers (**Supplementary Table 2**). An AlkPhos Direct Labelling Module and Detection System with CDP-Star Detection Reagent (GE Healthcare) were used. Bands were detected with an ImageQuant LAS 4000 mini camera system (GE Healthcare). pZAAMP-CTB-10Li45GB3A was used as a positive control, and WT rice genomic DNA was used as a negative control.

### Production of MucoRice-CTB 19A

**Figure 1:**
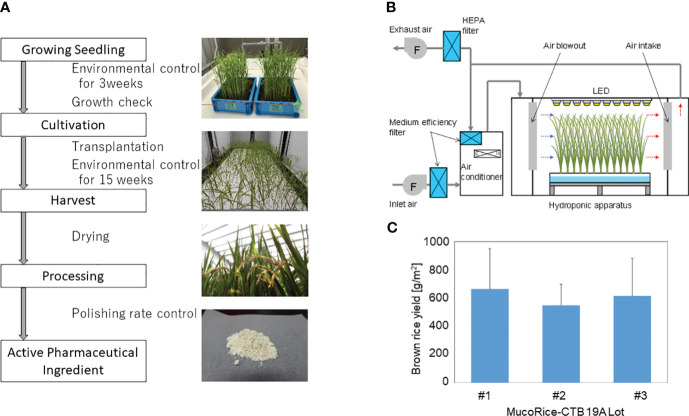
MucoRice-CTB line 19A production using a LED-based hydroponic system. **(A)** Flowchart of the manufacturing process from cultivation of MucoRice-CTB19A to rice powder, along with representative photographs at each stage. Key points of are indicated between blocks of the flowchart. **(B)** Schematic diagram of the HVAC system regulating the cultivation chamber environment. Horizontal airflow is generated within the hydroponic cultivation setup; controlled fresh air intake and exhaust maintain circulation of conditioned air within the system. **(C)** Brown rice yield of MucoRice-CTB19A, demonstrating three consecutive harvests. The number of plants analyzed for each lot was six.

MucoRice-CTB 19A was produced from master seed bank seeds in a closed plant production system at Chiba University. The production process flow from seed to rice flour (or active pharmaceutical ingredient) is shown in **Figure 1A**. Cultivation was performed by hydroponics using recirculating nutrient solution and polystyrene foam trays. Seeds of MucoRice-CTB 19A were sterilized by immersion in 70% ethanol followed by 1.25% sodium hypochlorite solution. These seeds were sown individually on a net attached to a square wooden frame at approximately 1.5 cm intervals and floated on water adjusted to pH 5.0 to 5.5. Liquid fertilizers (OAT House Fertilizers 1, 2, and 5; OAT Agrio Co., Ltd., Tokyo, Japan) were added five days after sowing. Seedlings were grown under automated control of temperature, humidity, and CO_2_ concentration and illuminated by multiple 40 W white LED lamps for 21 days. Seedlings were selected based on plant height and leaf number and transplanted at a density of approximately 200 plants/m^2^. Transplanted rice plants were grown under an automated controlled environment for approximately three months. **Supplementary Table 3** provides details on the settings for the seedling and cultivation environments, and **Figure 1B** shows the schematic diagram of the cultivation. Fertilizers were added to the nutrient solution according to the water supply, and the pH was controlled to be below 6.0 by the automated addition of pH adjusting reagent (OAT Agrio Co., Ltd.). The panicles were harvested at least 40 days after heading, dried in a well-ventilated room until the moisture content of the grains was sufficiently reduced, and threshed. Hulls were removed manually using a husker. Extracted brown rice was polished in a rice polisher (Kett Electric Laboratory Co. Ltd., Tokyo, Japan) to achieve a polishing rate of 95% and ground into a coarse powder in a mill (Iwatani Corporation, Osaka, Japan) to produce rice flour. To prevent the spread of genetically modified plants, exhaust air from the cultivation room was passed through HEPA filters to collect particulate matter. Waste nutrient solution was collected in a waste water tank. Condensed water from the air conditioner was either reused for humidification or discharged to the effluent tank. Water in the holding tank was automatically autoclaved and disposed of.

### Whole-genome re-sequencing

Genomic DNA was extracted and sequenced in a HiSeq2000 platform (Illumina) in 2016 as described (Mejima et al., 2015). Paired-end read sequences of 100 bp per read (Sanger FASTQ format) from both sides of each fragment were obtained using Casava software (ver. 1.13.48; Illumina).

### Determination of the position and sequence of the insertion

The flanking regions of each integrated sequence were determined as described (Mejima et al., 2015). Sequences LB-1 to -3 located near the left border (LB) and RB-1 to -4 near the right border (RB) (**Supplementary Table 4**) in the T-DNA vector were used to identify reads containing insert-specific sequences. The matched reads were mapped onto the insert construct by using the CLC Workbench 6.0 program (CLC bio, Aarhus, Denmark), and reads with insert sequences only were excluded. The remaining read sequences were subjected to National Center for Biotechnology Information (NCBI) BLAST (Oryza Blast) to estimate the insert position in the genome. Based on this information, primers (**Supplementary Table 2**) were designed for amplification, isolation, and cloning of the DNA, and the sequence of the genome flanking DNA and the entire sequence of the inserted DNA were determined.

### Full-length sequence analysis of the transgenes and its genomic flanking regions

Sequences of the cloned PCR products, including the genomic flanking region of chromosome 1 and the entire insertion region, were determined with Genetic Analyzer 3500 and compared to the WT Nipponbare rice genome using Genetyx software Oryza Blast (Genetyx, Tokyo, Japan).

### Shotgun MS/MS analysis

Salt-soluble proteins from mature brown seed of MucoRice-CTB 19A and WT (Nipponbare) were extracted by using 1 M NaCl. Shotgun proteomic analysis of peptide mixtures was performed as described in Kurokawa et al. (2013) using a nanoflow LC system (Dina-2A, KYA Technologies, Tokyo, Japan) in combination with a linear ion trap Orbitrap Mass spectrometer (LTQ-Orbitrap Velos, Thermo Fisher Scientific). Protein identification was performed by searching MS and MS/MS data against the NCBI nonredundant rice protein database using Mascot (Matrix Science). Decoy database searches were also performed using Mascot and filters were applied to meet a false positive rate of less than 1%.

### Protein analyses

Mature seeds of MucoRice-CTB 19A were harvested, and total seed protein was extracted and subjected to SDS-PAGE followed by Western blot analysis as previously described (Mejima et al., 2015). CTB levels in MucoRice-CTB were determined by SDS-PAGE densitometric analysis (Yuki et al., 2013). Briefly, SDS-PAGE gels were stained with Simply Blue Safe Stain kit (Invitrogen Life Technologies) for 12 h at room temperature, and the stained spot intensity of CTB on SDS-PAGE was determined on a GS-900 calibrated densitometer (Bio-Rad Laboratories, Tokyo, Japan) using a Bio-Rad Quantity One software. Because the CTB sequence of MucoRice CTB was authentic except for the amino acid change to Gln(Q) and the addition of the N-terminal S(Ser)-R(Arg) peptide, recombinant SR-CTB (N4Q) serial dilutions (0.2, 0.4, 0.8, and 1.6 μg) were used as standards for densitometric analysis (Yuki et al., 2013).

### Immunofluorescence microscopy

Mature seeds of MucoRice-CTB 19A were cut into semi-thin sections (0.5 μm) on an ultramicrotome (EM UC6, Leica, Wetzlar, Germany) as previously described (Kurokawa et al., 2014). For double staining of prolamin and glutelin, sections were incubated with rabbit anti-13 kDa prolamin antibody (1:1000) or mouse anti-glutelin A monoclonal antibody (5 μg/mL) for 1 h after blocking with 1% BSA-PBS. Then, sections were incubated with Alexa647-conjugated anti-rabbit IgG antibody (1:200) or DyLight488-conjugated anti-mouse IgG antibody (1:200) in 1% BSA-PBS for 1 h. For double staining of CTB and glutelin, sections were incubated with rabbit anti-CTB antibody (10 μg/mL) or mouse anti-glutelin A monoclonal antibody (5 μg/mL) for 1 h after blocking with 1% BSA-PBS. Then, sections were incubated with Alexa647-conjugated anti-rabbit IgG antibody (1:200) or DyLight488-conjugated anti-mouse IgG antibody (1:200) in 1% BSA-PBS for 1 h. Images were captured by a confocal laser scanning microscope (LSM 800 Axio Observer, Carl Zeiss, Oberkochen, Germany).

### Oral immunization

Six- to eight-week-old female BALB/c mice (CLEA, Tokyo, Japan) were used for immunization with MucoRice-CTB 19A. Mice were maintained at 22°C under a standard 12-h light/12-h dark cycle. Food and water were provided ad libitum. All experiments were performed in accordance with the “Guidelines for the Use and Care of Laboratory Animals” and approved by the Animal Committee of the Chiba University Institute of Medical Science. WT or MucoRice-CTB line 19A powder (150 mg containing 740 μg of CTB) suspended in 750 μL PBS was used for oral immunization by gastric intubation four times at 2-week intervals. Serum and fecal extracts were collected 1 week after the last immunization.

### ELISA

The CTB-specific ELISA was performed as described previously (Nochi et al., 2007).

Briefly, a 2-fold serial dilution of serum and nasal wash solution was added to the well of a 96-well microplate coated with 100 μL of 5 μg/mL CTB and incubated for 2 h at room temperature. After washing, HRP-conjugated goat anti-mouse IgG or HRP-conjugated goat anti-mouse IgA (Southern Biotechnology Associates, Birmingham, AL, USA) diluted 1:4000 was added and the plates were incubated for 1.5 h at room temperature. After developing by a TMB Peroxidase Substrate kit (XPL, Caithersburg, MD, USA), the endpoint titer was expressed as the reciprocal of the log2 of the last dilution that gave an OD_450_ 0.1 greater than the negative control.

### Toxin challenge

*In vivo* challenge experiments with CT were performed as described previously (Tokuhara et al., 2010). Briefly, vaccinated mice (10 mice per group) were orally administered 20 μg CT (List Biological Laboratories, Campbell, CA, USA); 12 h later, mice were euthanized and their small intestine and colon were removed for clinical evaluation of diarrhea and collection of intestinal contents. Samples were centrifuged and intestinal water content was measured.

### Data analysis

Group differences in data were evaluated using the two-tailed Student t test; a *P* value of less than 0.01 was considered statistically significant.

## Results

### Selection of MucoRice-CTB line 19A

Seeds of four marker-free MucoRice-CTB lines MucoRice-CTB 9B, 19A, 43B, and 50B, and WT Nipponbare were germinated and seedlings were planted in soil under sunlight in a greenhouse. Average fertility and number of seeds per plant in these lines relative to WT Nipponbare are shown in **Supplementary Figure 2**; the values of both parameters were higher in 19A than in the other lines.

In Southern blot analysis, hybridization with the *CTB* probe detected one EcoRI fragment (ca. 9 kb) and one SacI fragment (ca. 10 kb) in line 19A (**Supplementary Figure 3**), indicating a single copy of the *CTB* gene in its genome. We detected three copies in line 9B, one in line 43B, and two in 50A. We selected line 19A as a new MucoRice-CTB line because of its higher fertility and number of seeds per plant under the conditions tested, and its genetic simplicity (single copy of the transgene), which we expected to be a stable producing line.

### Production of MucoRice-CTB line 19A in a LED-based hydroponic system

To evaluate production stability, three consecutive cultivation experiments were conducted using the master seed bank. The results of the germination rate and pre-transplant growth assessment demonstrated the viability of the seeds and the uniformity of the environment for seedling growth (data not shown). The rice flour or active pharmaceutical ingredient used for analysis was obtained according to the process flowchart shown in **Figure 1A**. The brown rice yield from each of the three cultivation rounds was calculated based on six randomly selected plants, respectively. The yields were either equal to or greater than 588.5 g/m^2^ (**Figure 1C**).

### Genomic and protein analyses of MucoRice-CTB line 19A

**Figure 2:**
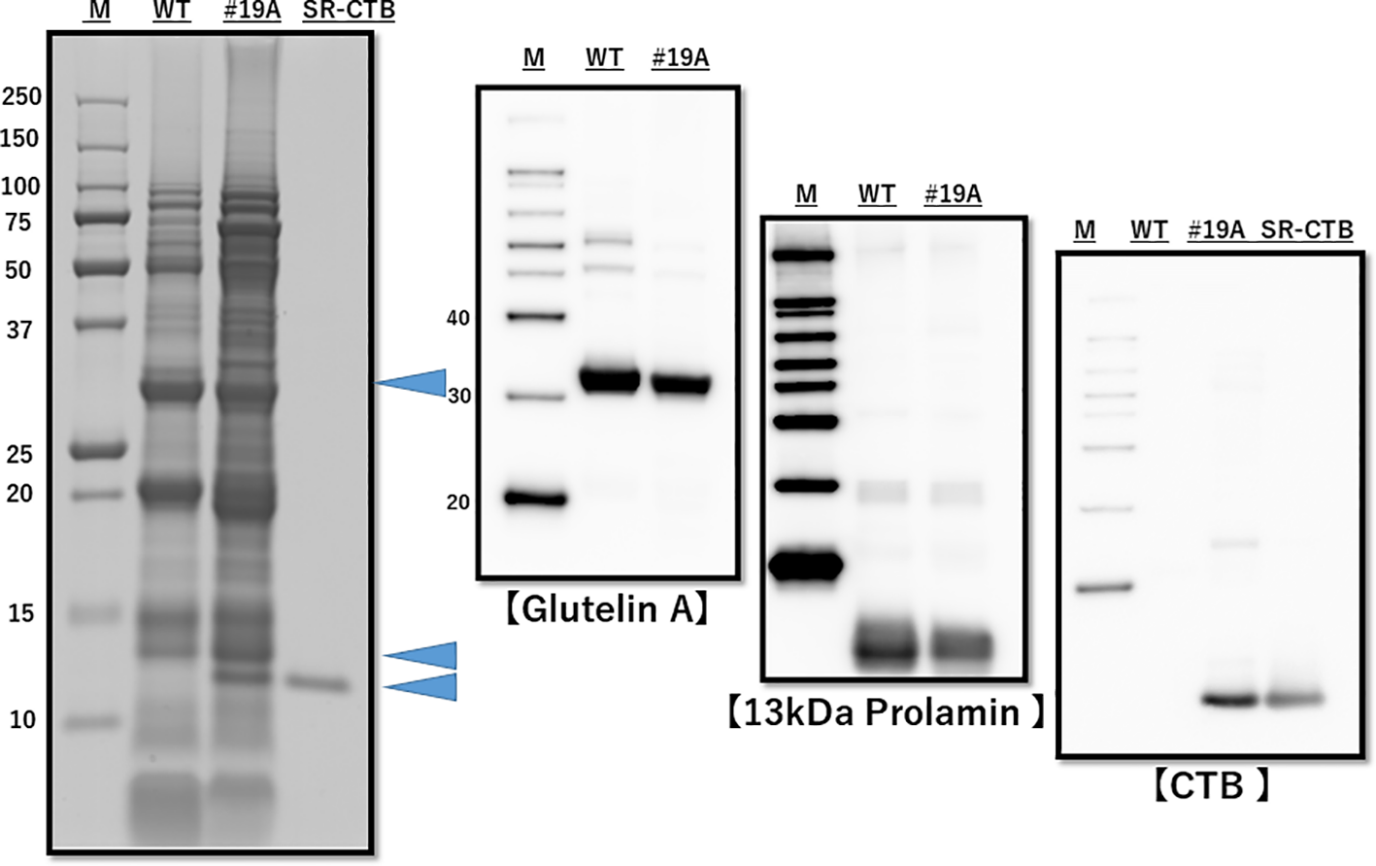
SDS-PAGE and western blot analysis of total protein from WT and MucoRice-CTB line 19A seed. Proteins were extracted with SDS-sample buffer, separated by SDS-PAGE (left panel), and western-blotted with the indicated antibodies (right panels). The levels of endogenous storage proteins such as glutelin A and 13-kDa prolamin were lower in MucoRice-CTB19A than in WT rice. M, molecular weight markers; SR-CTB, recombinant CTB.

The presence of the *CTB* gene and absence of the *HPT* gene were confirmed by genomic PCR (**Supplementary Figure 4**), and the presence of CTB proteins was confirmed by western blot analysis (**Figure 2**). The endosperm proteins glutelin A and 13K prolamin were downregulated (**Figure 2**). Downregulation of mRNA of glutelins A and B and 13K prolamin by RNAi was confirmed by quantitative real-time PCR (**Supplementary Figure 5**). SDS-PAGE densitometric analysis (n=3 lot) showed that the CTB protein level was 4.94 ± 0.29 μg/mg in line 19A and 6.52 ± 0.22 μg/mg in line 51A.

### Genomic location and structure of the transgenes in MucoRice-CTB line 19A

**Figure 3:**
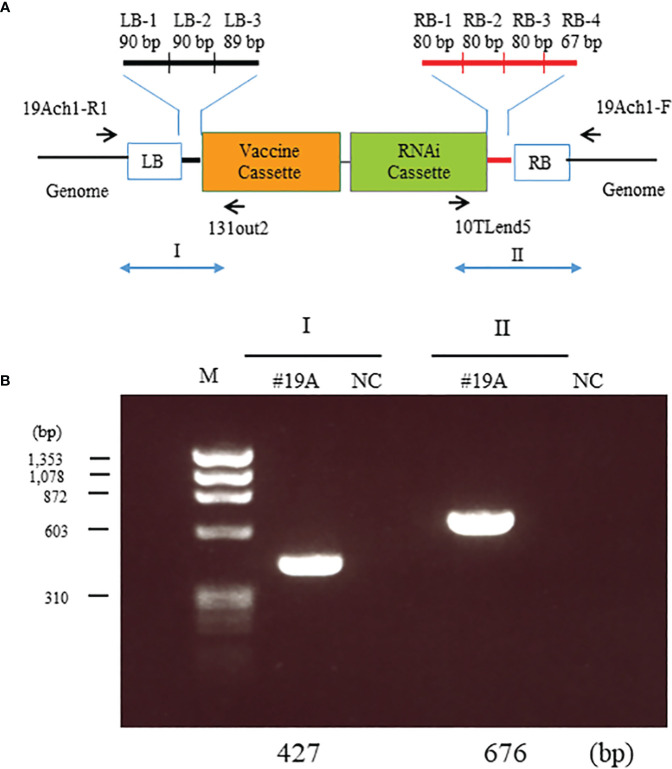
Identification of T-DNA insertion sites in MucoRice-CTB line 19A. **(A)** Location of the seven insertion-specific sequences (left border [LB]-1, -2, and - 3; right border [RB]-1, -2, -3, and -4) used to identify T-DNA flanking sequences in the whole genome resequencing analysis. Arrows mark the positions of primers used to identify T-DNA flanking sequences in PCR analysis. **(B)** PCR analysis to detect T-DNA flanking sequences on chromosomes 1. Genomic DNA from a homozygous MucoRice-CTB line (#19A) was used as a template with the indicated primer pairs (**Supplementary Table 1**). Sizes of the PCR products are indicated below the gel. M, DNA size marker; NC, no template.

**Figure 4:**
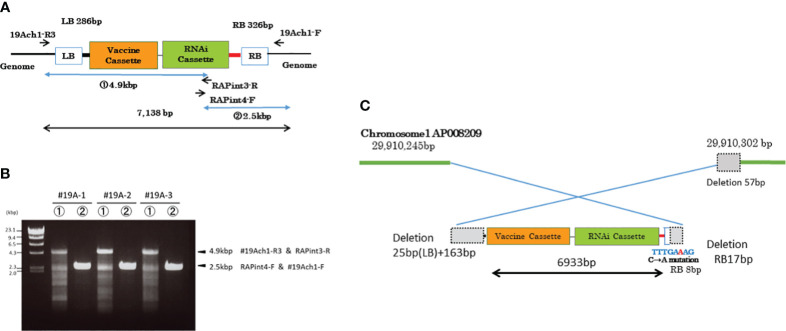
DNA insertion in chromosome 1 of MucoRice-CTB line 19A. **(A)** T-DNA vector design. Positions of PCR primers ae indicated. **(B)** PCR amplification of inserted DNA with the indicated primers. The PCR products were cloned and their full sequences were determined. **(C)** Schematic diagram showing the nucleotide sequences at the junctions between the T-DNA sequence and the two flanking regions of chromosome 1. The genomic and T-DNA sequences deleted during the insertion process are shown as dotted gray squares. The truncated right border (RB) of the T-DNA is shown as a box with blue outline. A single copy of the transgene was inserted into chromosome 1 in an inverted orientation.

To investigate the location of the transgenes, we resequenced the whole genome of MucoRice-CTB line 19A; the data are available in the DDBJ and NCBI Sequenced Read Archive under the accession number SAMD00491847 and DRX362635, respectively. We determined the position of the insertion by identifying reads containing sequences near the RB or LB sequences of the T-DNA (**Figure 3A**) and by a BLASTN search of these sequences against the Nipponbare genome. The position of the insertion was 29,910,245 bp on chromosome 1. We also amplified the flanking regions in MucoRice-CTB line 19A with the primer pairs 19Ach1-R1 and 131out2 for LB, and 10TLend5 and 19Ach1-F for RB (**Figures 3A, B**; **Supplementary Table 2**). Sequencing of the PCR products showed a 57-bp deletion corresponding to 29,910,246–29,910,302 bp on chromosome 1 of the WT rice genome (**Figure 4C**).

To clarify structure of the transgene, we amplified the inserted sequence in MucoRice-CTB line 19A chromosome 1 with the primer pairs 19Ach1 R3 and RAPint3-R, and RAPint4-F and 19Ach1-F (**Figure 4A**; **Supplementary Table 2**), and cloned and sequenced it the PCR products. We demonstrated that a single copy of the transgene (6933 bp) was inserted into chromosome 1 in the reverse orientation (**Figure 4C**; **Supplementary Figure 6**). To avoid PCR errors, we carried out PCR and cloning three times (**Figure 4B**). In comparison with the T-DNA binary vector, the inserted sequence showed (1) a deletion of the entire LB 25 bp and its downstream region (163 bp), (2) a 17-bp deletion from the 3′-end of the RB and a C-to-A point mutation in one of remaining 8 bp of RB (**Figure 4C**). One copy each of the complete *CTB* gene cassette and RNAi cassette were consistent with the results of the Southern blot analysis (**Supplementary Figure 3**).

### Location of CTB and storage proteins analyzed by immunofluorescence

**Figure 5:**
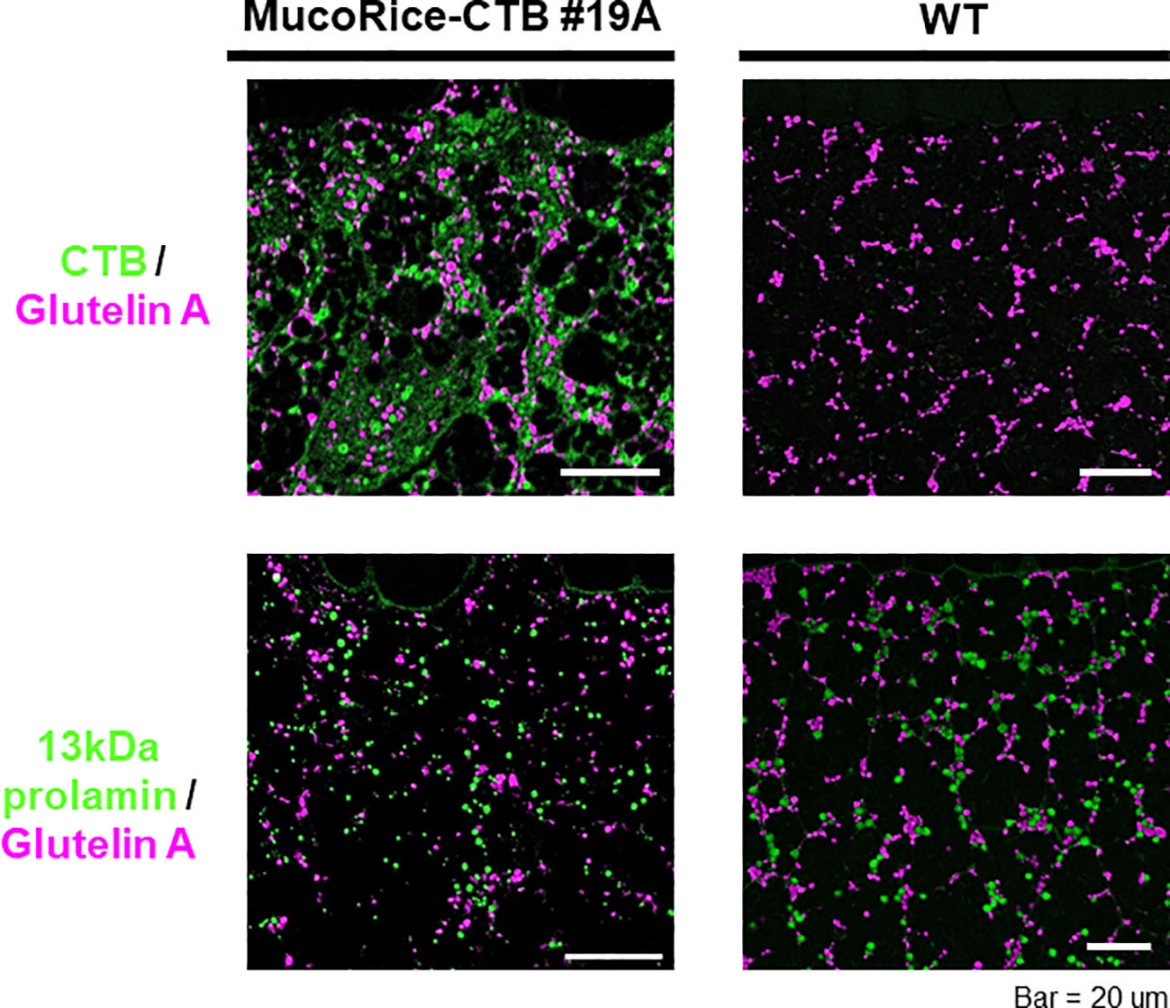
Immunofluorescence analysis of storage proteins in WT and MucoRice-CTB line 19A seeds. Double labeling was performed with the mouse monoclonal antibody against glutelin A (pink) and rabbit polyclonal antibody against CTB or 13-kDa prolamin (green). Scale bars, 20 µm.

In MucoRice-CTB line 19A, the CTB signal was most abundant in the subaleurone layer and was absent in the aleurone layer (data not shown). The intensity of the CTB signal decreased toward the interior of the seed as the protein granules decreased and starch grains dominated (data not shown). CTB was prominent in the cell wall, cytoplasm, in protein bodies (PB)-I and -II, although the total amount of CTB was small (**Figure 5**). The number and size of PB-I (stained with 13-kDa prolamin antibody) and PB-II (stained with glutelin A antibody) appeared to be lower in MucoRice-CTB than in WT (**Figure 5**), as expected from downregulation of these storage proteins by RNAi.

### Shotgun MS/MS proteomics of MucoRice line 19A

The CTB (N4Q) protein was present in MucoRice-CTB 19A, with a sequence coverage of 85% (**Supplementary Figure 7**), but not in WT. The number of identified salt-soluble proteins was 452 in MucoRice-CTB line 19A and 405 in WT rice, with an overlap of 254 proteins (**Supplementary Table 5**). The total number of matching peptides in the MS/MS spectra was then calculated for each protein to obtain the peptide spectral match (PSM). Since PSM is proportional to the amount of a protein (Liu et al., 2004), the relative amount of MucoRice-CTB line 19A proteins overlapping with WT proteins was calculated as the PSM ratio, 19A/WT. The major identified rice allergens and their PSM ratios are listed in **Table 1**. The levels of 63-kDa globulins, glyoxalase I, and α-amylase/trypsin-like protein family such as RAG2, RA5R and RA16, were lower in line 19A than in WT seeds, whereas the levels of other allergens, 19-kDa and 52-kDa globulins, did not differ. These results suggest that line 19A raises fewer safety concerns than WT rice.

**Table 1:** Allergenic proteins differentially expressed in MucoRice-CTB 19A.

Accession number	Description	Allergen name	Theoretical MW(kDa)/pI	Expression ratio MucoRice-CTB/ WT
Q75GX9.1	63 kDa globulin-like protein	63kDa globulin	63.4/8.13	0.825
XP_015628337.1	cupincin	52kDa globulin	52.1/7.25	1.078
BAB71741.1	glyoxalase I	Glyoxalase I	32.5 / 5.67	0.446
AAA72362.1	unnamed protein product	19kDa globulin	19.8 / 6.96	0.991
ACA50505.1	seed allergenic protein RAG2	RAG2	17.8 / 8.03	0.114
XP_015646664.1	seed allergenic protein RAG2-like		17.3 / 8.34	0.133
Q01881.2	Seed allergenic protein RA5	RA5	17.3 / 8.03	0.719
XP_015645223.1	alpha-amylase/trypsin inhibitor RA16	RA16	17.0 / 8.03	0.383

### Protective immunity against CT-induced diarrhea by oral immunization with MucoRice-CTB line 19A

**Figure 6:**
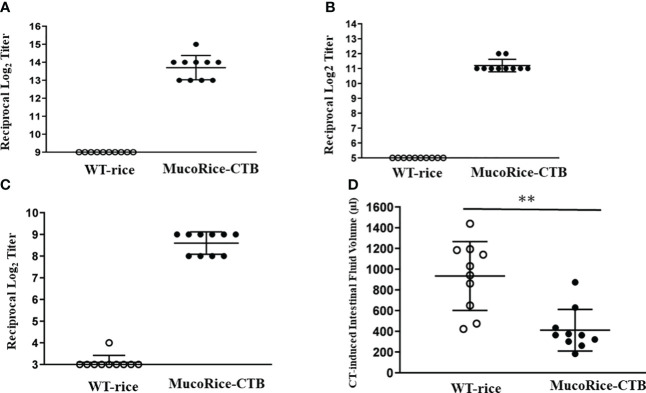
Antibody responses and protective immunity induced by oral immunization with MucoRice-CTB line 19A. **(A–C)** Mice were immunized 5 times at 2-week intervals with MucoRice-CTB line 19A or WT rice powder suspended in PBS. CTB-specific serum IgG **(A)**, serum IgA **(B)**, and fecal IgA **(C)** responses were induced in mice immunized with MucoRice-CTB line 19A, but not with WT rice. **(D)** Mice vaccinated with MucoRice-CTB line 19A had lower intestinal water content than mice that received WT rice. **P < 0.001.

In order to determine whether MucoRice-CTB line19A could induce antigen-specific mucosal immune responses, mice were orally immunized with unpurified polished flour of line 19A or WT rice. MucoRice-CTB line 19A, but not WT rice, induced serum CTB-specific IgG and IgA and fecal CTB-specific IgA immune responses (**Figures 6A–C**). These mice were challenged orally with CT. Mice immunized with MucoRice-CTB were protected from CT-induced diarrhea, since mice immunized with the same amount of WT rice flour developed severe diarrhea, as evidenced by a significantly higher intestinal fluid volume (**Figure 6D**). To evaluate the protective efficacy, we measured the intestinal fluid volume in immunized mice without CT challenge in a control experiment (**Supplementary Figure 8**). We found a significant difference (*P* = 0.0469) between the control mice (267 ± 74 μL) and CT-treated mice (411 ± 201 μL). These results indicate that MucoRice-CTB strain 19A has high oral immunogenicity and is capable of inducing protective immunity.

## Discussion

In this study, we selected MucoRice-CTB line 19A from previously established marker-free lines as a new master seed bank for the stable production of transgenic rice–based cholera vaccine. Although Phase I studies revealed no problems with safety, tolerability, or immunogenicity of MucoRice-CTB line 51A in humans in Japan and the U.S.A., we failed to obtain sufficient yield for Phase II because the MucoRice-CTB line 51A did not develop under strong light such as sunlight. MucoRice-CTB line 51A had a tandem copy of the transgene on chromosome 3 and a partial sequence on chromosome 12 outside an exon (Mejima et al., 2015), whereas the 19A strain had a single complete copy of the transgene on chromosome 1.

First, we compared fertility and seed number in the soil in sunlight of four marker-free strains produced in 2015. MucoRice-CTB line 19A had approximately the same average fertility and seed number as WT Nipponbare. Line 43B also had just one copy of the transgene but its average fertility and seed number were lower, suggesting a problem in transgene position. In MucoRice-CTB line 19A, the insertion site of the transgene was located at 29,910,245 bp on chromosome 1 in an intergenic region (**Figure 4C**), 17,342 bp downstream of a gene whose product is similar to systemin receptor SR160 precursor (Os01g0718300; 29,927,587–29,931,452), and 541 bp upstream of the gene for isoleucyl-tRNA synthetase (Os01g0718150; 29,907,349–29,909,704). In the characterization of MucoRice-CTB 19A master seed bank, a PCR test of the LB and RB flanking regions of chromosome 1 (**Figure 3**) will be a powerful tool to confirm the genetical stability of this line. We determined the DNA sequence of the transgene of MucoRice-CTB line 19A (**Supplementary Figure 6**); this information will be used when producing the next seed bank. Although the master seed bank can be preserved in the cold for at least 10 years (Sasou et al., 2021), we will need to produce the next seed bank from the master seed bank in near future.

For stable supply of a large quantity of MucoRice, we aimed to establish a low-cost MucoRice-CTB cultivation system that uses the latest light source, LED (Jähne et al., 2020). In closed hydroponics with LED, the 19A line achieved seed yields (**Figure 1**) equal to or better than those of the 51A line (average 500 g/m^2^) at the same light level (600–700 μmol/m^2^ s) under a metal halide lamp (Kashima et al., 2016). Line 19A also developed well in sunlight, and the most recent data with doubled light intensity (1400 μmol/m^2^ s) yielded more than 1000 g/m^2^ (data not shown); line 51A cannot grow in this condition. The CTB content of 19A per powder weight was about 75% of that of 51A, which was significantly lower, and thus does not strongly depend on the *CTB* gene copy number. We confirmed that oral immunization of mice with rice powder of MucoRice-CTB line 19A induced protective immunity with antigen-specific systemic and mucosal antibody immune responses (**Figure 6**) in the same conditions as in our previous CT challenge test with MucoRice-CTB 51A (Mejima et al., 2015).

MucoRice-CTB has been developed as a uniform rice-derived oral cholera vaccine using an overexpression system of CTB (N4Q), a modified cholera toxin B subunit, and RNAi to inhibit the production of key rice endogenous storage proteins to increase CTB expression (Yuki et al., 2013). An increase in the level of the heat stock protein Bip has been reported in MucoRice-CTB line 51A (Kurokawa et al., 2013, Kurokawa et al., 2014). Among soluble rice proteins, we detected 70-kDa Bip1 (accession #XP_015625618.1) in our MS/MS data, with the highest PSM score (19A/WT, 37.667; **Supplementary Table 5**). We have also detected Bip as a 75-kDa protein band by western blotting that was substantially stronger in MucoRice-CTB line 19A than in wild-type rice (**Supplementary Figure 9**). The MS/MS analysis also confirmed the CTB sequence, including a N4Q substitution, in MucoRice-CTB 19A (**Supplementary Figure 7**). The levels of glutelin A/B and 13K prolamin were lower in MucoRice-CTB 19A than in WT in real-time PCR analyses (**Supplementary Figure 5**) and SDS-PAGE analysis (**Figure 2**). This also resulted in a down-regulation of PB-I (stained with 13 kDa prolamin antibody) and PB-II (stained with glutelin A antibody) in MucoRice-CTB line 19A than in WT seed in immunofluorescence analysis (**Figure 5**), consistent with the observations in MucoRice-CTB line 51A (Kurokawa et al., 2014).

In spite of the widespread consumption of rice in the world, rice allergy is a rare disease (Trcka et al., 2012). MucoRice-CTB has been developed as purification-free oral vaccine; thus, one of the safety concerns is the absence of elevated levels of known rice allergens in MucoRice-CTB (Goodman et al., 2008). Five major rice allergens have been identified, namely RAG2, a member of the α-amylase/trypsin inhibitor-like family (Adachi et al., 1993), 33-kDa glyoxalase I (Usui et al., 2001), 19-kDa globulin, and 52- and 63-kDa globulin-like proteins (Satoh et al., 2011). Proteomics approach revealed that that the RAG2 level was lower in MucoRice-CTB than in WT rice (Kurokawa et al., 2013). In MucoRice-ARP1, which produces a fragment of llama heavy chain antibody to rotavirus (ARP1), these allergens were not affected (Yuki et al., 2016). In MucoRice-CTB, RAG2 competed with CTB and was down-regulated (Kurokawa et al., 2014). In the present study, shotgun MS/MS analysis of proteins solubilized with 1 M salt showed that the levels of α-amylase/trypsin inhibitor-like protein family were decreased in MucoRice-CTB 19A (**Table 1**), suggesting that allergenicity is less of a concern with this line than with WT rice.

A dose-escalation Phase I study will be conducted in healthy male and female subjects at Chiba University Hospital and will be completed by 2026. In addition to the safety and tolerability of up to 18 g of the investigational drug (rice flour), this trial will ensure its efficacy against cholera by demonstrating the presence of oral immune response–inducing serum and saliva toxin-specific IgA antibodies, and toxin-specific secretory IgA induced in the intestinal tract, which has not been demonstrated in previous trials (Yuki et al., 2021, Yuki et al., 2022). In the near future, we plan to conduct challenges with *V. cholerae* and/or enterotoxigenic *E. coli* in humans to demonstrate that MucoRice-CTB line 19A protects against cholera and traveler’s diarrhea.

## Data availability statement

The datasets presented in this study can be found in online repositories. The names of the repository/repositories and accession number(s) can be found below: NCBI SRA, accession number: DRX362635 available at https://ddbj.nig.ac.jp/resource/biosample/SAMD00491847, and https://www.ncbi.nlm.nih.gov/sra/DRX362635.

## Ethics statement

The animal study was approved by the Institutional Animal Care and Use Committee of Chiba University. The study was conducted in accordance with the local legislation and institutional requirements.

## Author contributions

YY: Resources, Validation, Writing – original draft, Formal analysis, Investigation, Project administration. SK: Data curation, Investigation, Writing – review & editing. KS: Data curation, Investigation, Writing – review & editing. KK: Data curation, Investigation, Resources, Software, Validation, Writing – original draft. SM: Data curation, Investigation, Writing – review & editing. TY: Data curation, Writing – review & editing. AH: Data curation, Investigation, Validation, Writing – review & editing. MM: Data curation, Investigation, Methodology, Validation, Writing – review & editing. NT: Data curation, Validation, Writing – review & editing. MK: Methodology, Validation, Writing – review & editing. HK-H: Data curation, Writing – review & editing. MO: Resources, Validation, Writing – review & editing. TM: Resources, Writing – review & editing. RN-O: Formal Analysis, Validation, Writing – review & editing. KF: Validation, Writing – review & editing. TH: Supervision, Validation, Writing – review & editing. EG: Investigation, Resources, Supervision, Validation, Writing – review & editing. HK: Funding acquisition, Investigation, Project administration, Writing – review & editing.
