# Supplementary material for: MucoRice-CTB line 19A, a new marker-free transgenic rice-based cholera vaccine produced in an LED-based hydroponic system
Source: Front Plant Sci. 2024 Mar 15;15:1342662. doi: 10.3389/fpls.2024.1342662 (PMC10978600; doi:10.3389/fpls.2024.1342662)
Supplement: Supplementary file 1 [file DataSheet_1.pdf]

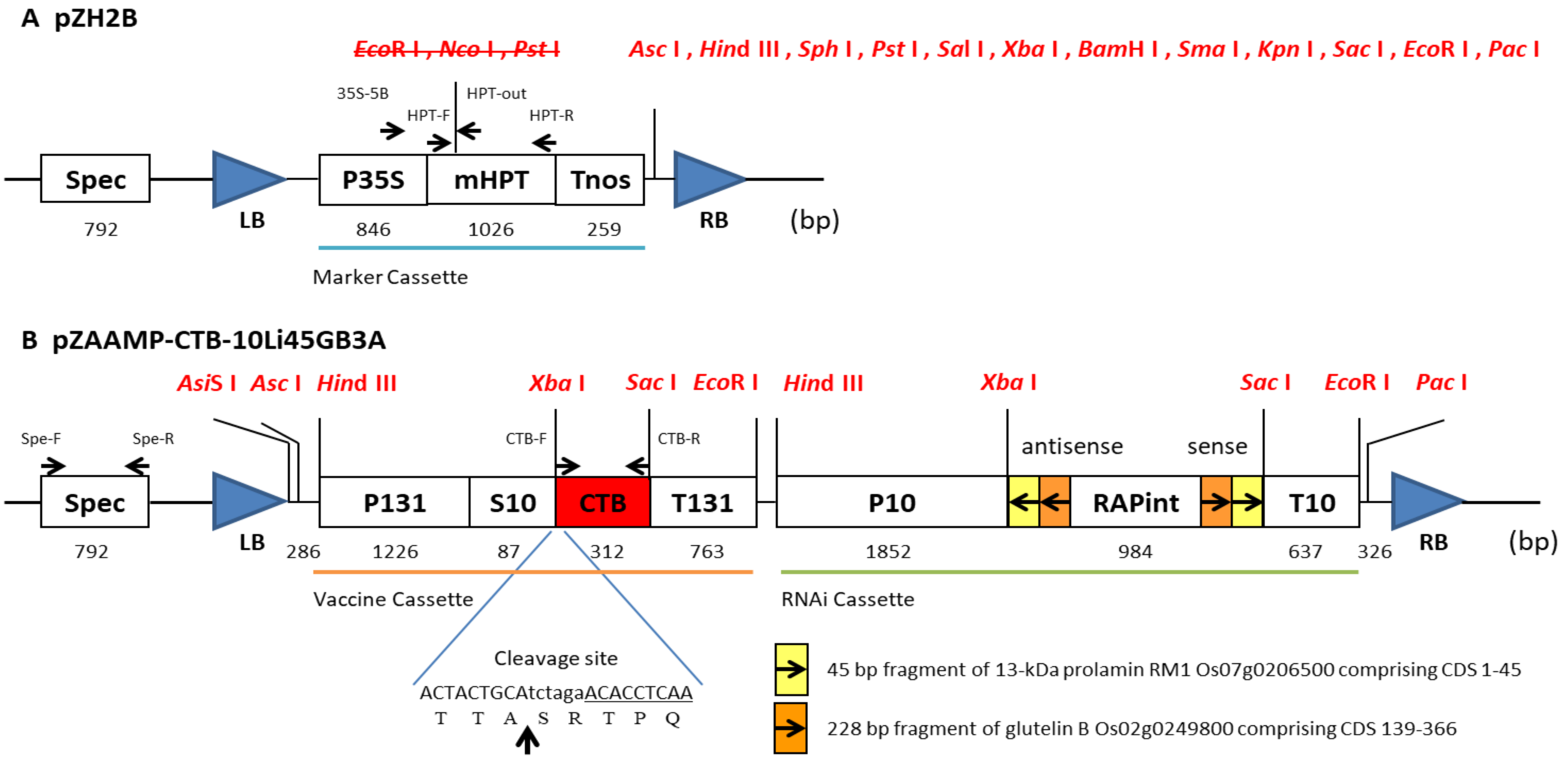

Supplementary Figure 1

T-DNA binary vectors used for Agrobacterium-mediated co-transformation. (A) Selection marker vector pZH2B containing an expression cassette for modified hygromycin phosphotransferase (*mHPT*; with deleted EcoRI, NcoI, and PstI sites) controlled by the cauliflower mosaic virus 35S promoter (P35S). Spec, spectinomycin resistance gene; LB, T-DNA left border; RB, T-DNA right border; Tnos, NOS terminator. (B) Selection marker–free *CTB*/RNAi vector (pZAAMP-CTB-10Li45GB3A) containing an overexpression cassette for *CTB*, controlled by the 13-kDa prolamin clone RM1 promoter (P131), and an RNAi cassette for suppression of rice endogenous storage proteins (13-kDa prolamin and glutelin B), controlled by the 10-kDa prolamin promoter (P10). The vertical arrow indicates the cleavage site between the prolamin signal sequence and CTB. S10, signal sequence of 10-kDa prolamin; CTB, cholera toxin B subunit; T131, 13-kDa prolamin clone RM1 terminator; RAPint, rice aspartic protease 3rd intron; T10, 10-kDa prolamin terminator.

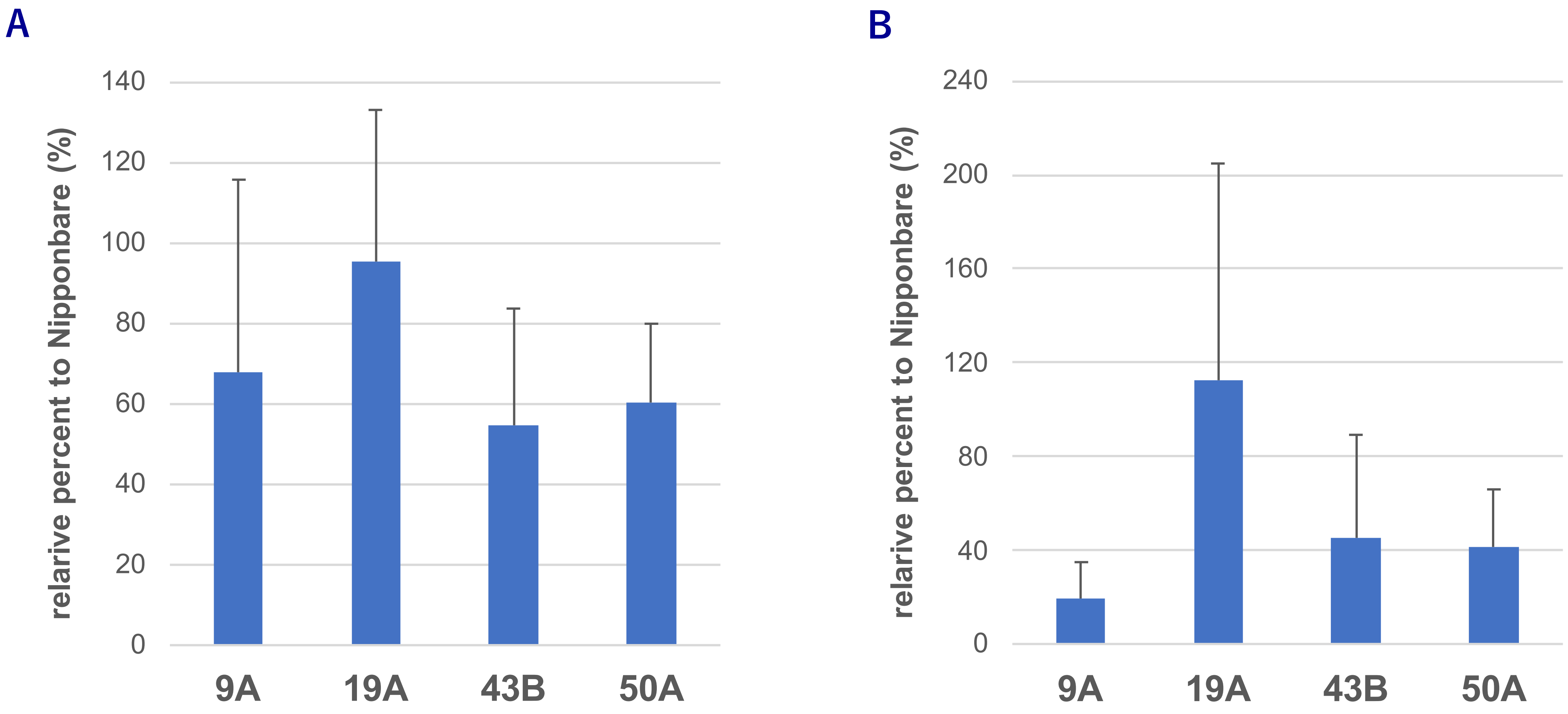

Supplementary Figure 2

Growth of *HPT* marker–free MucoRice-CTB lines 9B, 19A, 43B, and 50B under sunlight. Seeds (n=18-25) were germinated and planted in soil under sunlight, and recombinant plants were compared to wild-type Nipponbare in a greenhouse; the data are expressed as percentage. (A) Average fertility. (B) Average number of seeds per plant.

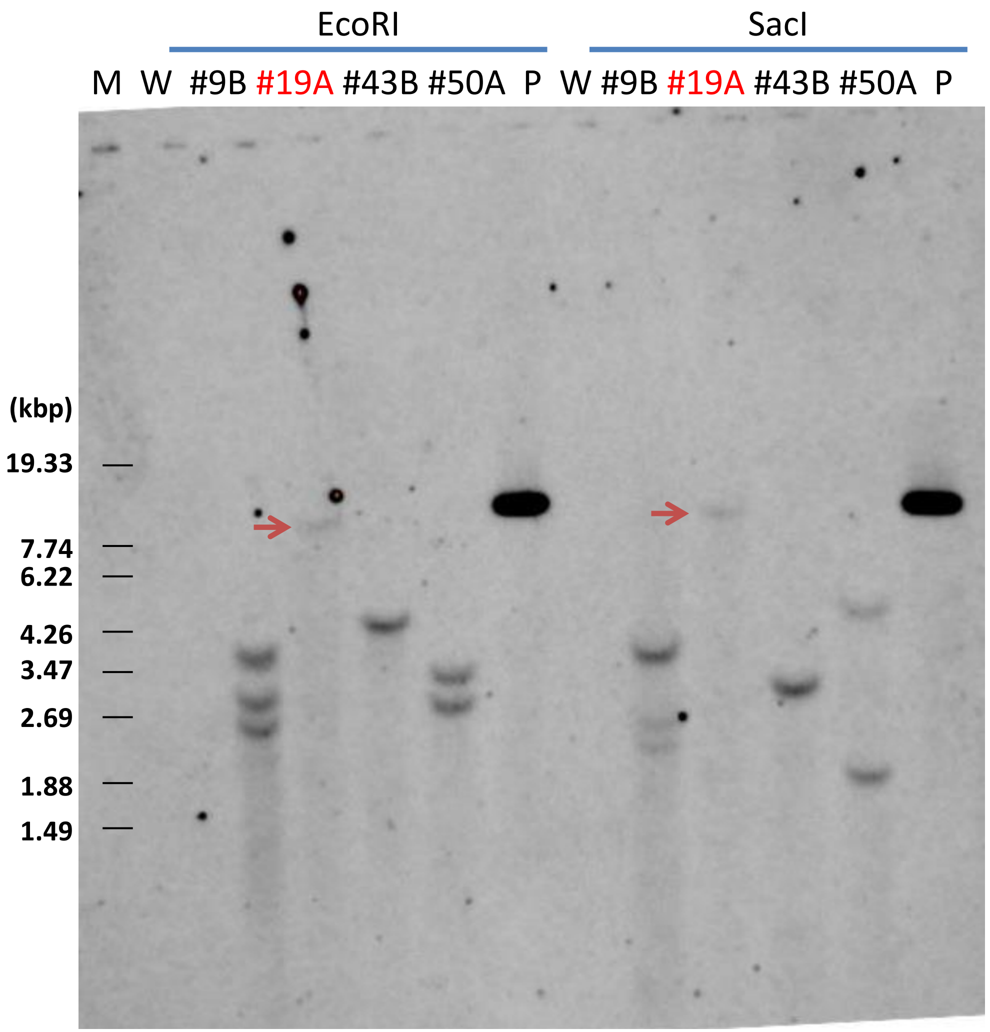

Supplementary Figure 3

Southern blot analysis of HPT marker–free MucoRice-CTB lines. Analysis was performed on genomic DNA of the fourth generation. The CTB probe detected a single band (arrows) in MucoRice-CTB line 19A, but no bands in wild-type Nipponbare. We inferred that a single copy of the CTB gene was present in the genome of the fourth generation of MucoRice-CTB #19A. The 9B, 43B, and 50A lines were estimated to have three, one, and two copies of the CTB gene, respectively. M: DNA size marker, W: wild-type Nipponbare, P: pZAAMP-CTB-10Li45GB3A Plasmid (2.5ng)

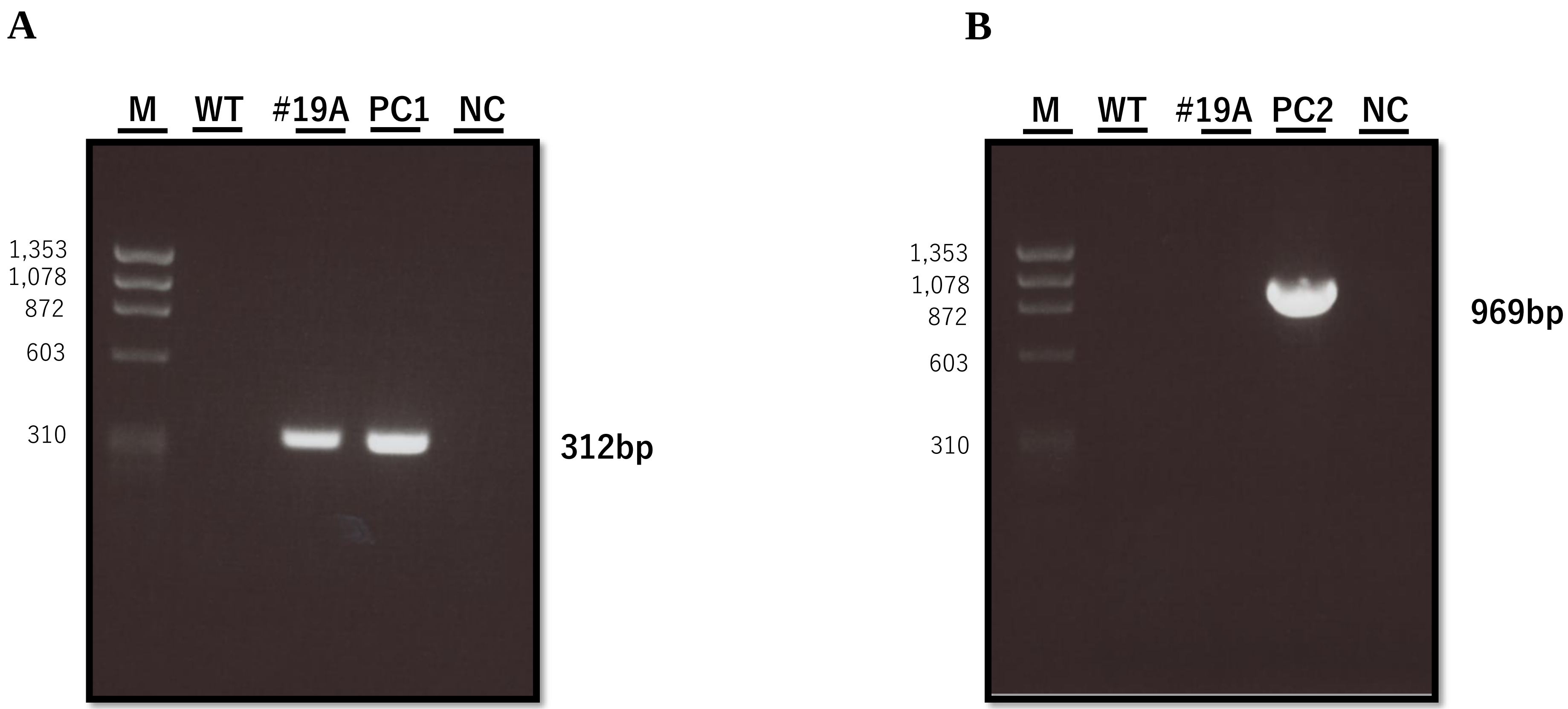

Supplementary Figure 4

Expression of CTB (A) and HPT (B) in MucoRice-CTB line 19A. Expression was analyzed by PCR. As controls, the following DNA templates were used: pZAAMP-CTB-10Li45GB3A plasmid (0.1 ng; PC1), pZH2B plasmid (0.1 ng; PC2), and genomic DNA from wild-type Nipponbare (WT). Primers are listed in Supplementary Table 1. M, DNA size marker; NC, no template.

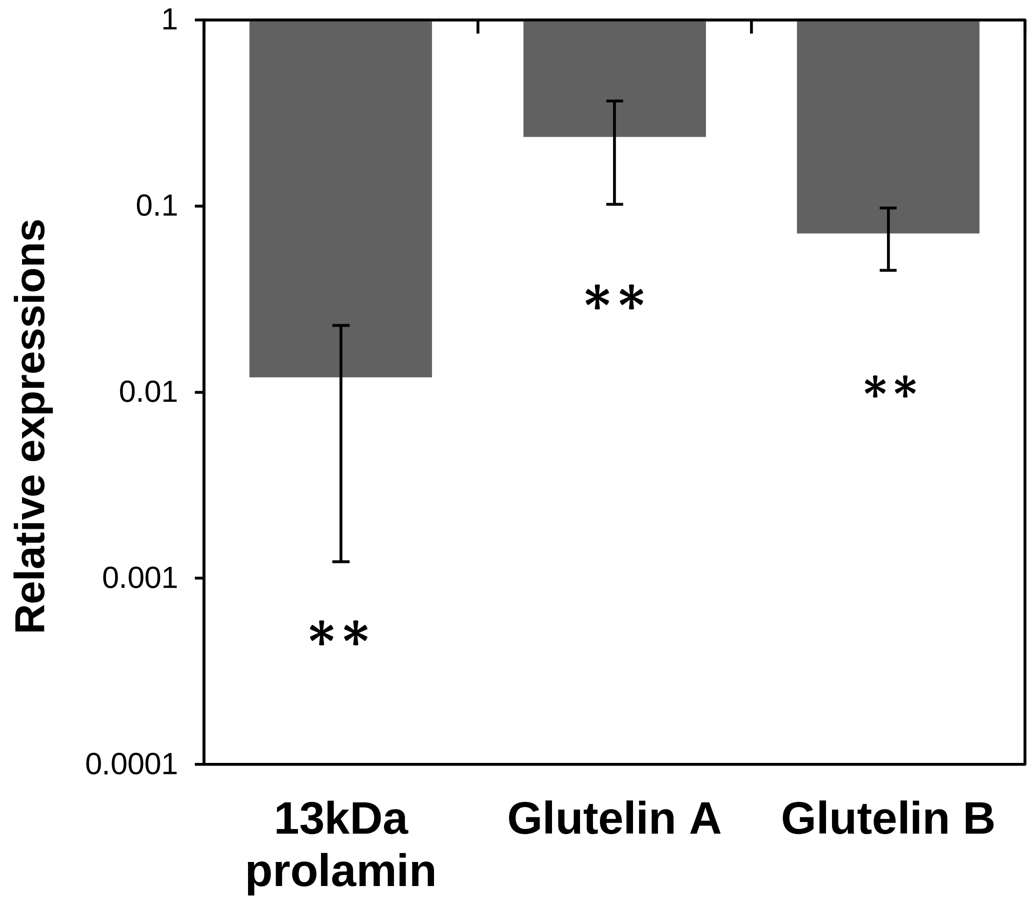

Supplementary Figure 5

mRNA levels of endogenous storage proteins in MucoRice-CTB line 19A. mRNA levels were analyzed by quantitative real-time PCR using RNA extracted from MucoRice-CTB line 19A or wild-type Nipponbare at 14 days after flowering. mRNA levels were normalized to the levels of 17S rRNA and are represented relative to those in wild-type Nipponbare. Values are averages of three replicates. ** $P < 0.01$ (MucoRice-CTB versus WT rice).

Supplementary Figure 6. Sequences of DNA insertion in Chromosome 1 of MucoRice-CTB 19A line

```
   1 TGCTGCAAGG CGATTAAGTT GGGTAACGCC AGGGTTTTCC CAGTCACGAC GTTGTAAAAC
  61 GACGGCCAGT GCCAAGCTGG CGATCGCTTT GGCGCGCCAA GCTTTTTGTC TAGTTGGCAG
 121 CCTAATTAAT TCTATGGAAA CCAGGTGACA TGGAGGGTTG GGGACATGGT GGAAAAAACC
 181 GGAACGGGCC GACAGTTCAA CCGGAAAAAA CCAGAACCCG TTCAGTTCAA AAGAAAGACC
 241 GGACATGCAT ATGACCCGCT TTGAACCGGC AGAACCGGTC GGTTTTTCTA TGAACCGGTC
 301 ATTAAACCGT CCCCGGTTAG ACCGAACAAG CCACAATAAT CTTGAAATGG GCCTTGATGT
 361 GGCCCAATTG GTCTGCCTAG AGCGTTTTGG TTGGCAAAAA TCAATCTCCT ATTCTCGGCA
 421 CGTGTGATAT ACAATGGTAA GTGAGATATA CAATTCTCGG CACGGCTACA TTACAAGGTG
 481 TCGCATTGTG TCAATGTTTG GTTAATTTGC TAGATTCACA TAATACATGC CAGGAAGTTC
 541 AGAACAATGT GTTGCCTTTC ACCGGAAAAC TTTGTTGGAG CAAATGCCTT CTTCTTTTTT
 601 GCTTCTGCTT CTTGAGTCCA TGTGGAGGAA GCAGTAGATA GCTGATGATA TCAGGATTCC
 661 TTCTGTGTCT GTGTAGGTGT AGCAACACCA CTATAATTTT TATTTAGCAA CACAATATCA
 721 ATTTGGTCTA TAAAAGTATG AATTAAATCA ATCCCCAACC ACAATTAGAG TAAGTTGGTG
 781 AGTTATTGTA AAGCTCTGCA AAGTTAATTT AAAAGTTATT GCATTAACTT ATTTCGTATC
 841 ACAAACAAGT TTTCACAAGA GTATTAATGG AACAATGAAA ACCATTGAAC ATACTATAAT
 901 TTTTTTTCTT ACTGAAATTA TATAATTCAA AGAGCATAAA CCCACACAGT CGTAAAGTTC
 961 CACGTGTAGT GCATTATCAA AATAATAGCT TACAAAACAT AACAAACTTA GTTTCAAAAG
1021 TTGCAATCCT TATCACATTG ACACATAAAG TGAGCGATGA GTCATGTCAT TATTTTTTTG
1081 CTCACCATCA TGTATATATG ATGGGCATAA AAGTTACTTT GATGATGATA TCAAAGAACA
1141 TTTTTAGGTG CACCTAACAG AATATCCAAA TAATATGACT CACTTAGATC CTAATATAGC
1201 ATCAAGCAAA ACTAACACTC TAAAGCAACC GATAGGGAAA CATCTATAAA TAGACAAGCA
```

1261 TAATGAAAAC CCTCCTCATC CTTCACACAA TTCAAACATT ATAGTTGAAG CATAGTAGTA
1321 GAATCCTACA AAATCTAGTA TTGTAGAATC AGCAATGGCA GCATACACCA GCAAGATCTT
1381 TGCCCTGTTT GCCTTAATTG CTCTTTCTGC AAGTGCCACT ACTGCATCTA GAACACCTCA
1441 ACAGATTACT GATTTGTGTG CAGAATACCA CAACACACAG ATCCACACCC TCAATGATAA
1501 GATTTTCTCT TATACAGAAT CTCTAGCTGG AAAGAGAGAG ATGGCTATCA TTACTTTCAA
1561 GAATGGTGCA ACTTTCCAAG TAGAAGTACC AGGCAGTCAA CATATAGATT CACAAAAGAA
1621 GGCAATTGAA AGGATGAAGG ATACCCTGAG GATTGCATAT CTTACTGAAG CTAAAGTTGA
1681 AAAGCTATGT GTATGGAATA ACAAGACTCC TCATGCAATT GCCGCAATTA GTATGGCAAA
1741 TTGAGAGCTC ATTGTAATAG TATAATGGTT CAAATGTTAA AAATAAAGTC ATGCATCATC
1801 ATGCGTGACA GTTGAAACTT GATGTCATAT AAATCTAAAT AAAATCACCT ATTTAAATAG
1861 CATTCATGTA TGAGGTTGCA TTATCATAGC TAATTACCAT CACAAAGAAC TTTACAATTA
1921 CTATGTGCAT GCATTTGATC CTAAGCTACT TTGGCTATTA GATACAAATG GAGTGTATTA
1981 AGCAAGTCCA ACTTTTCATT CTAATAGGAA CAAACTTGAA GCACATATGT CAATTACTAT
2041 ATCCCCAACA ATTGATACAC TATAGCAGTG GTTGCAATGG CAGCTGCAGG AATTGCTAGT
2101 GAACCCAACA GCCCATATAA CTGAATTGAA GCCATGTCAG ACATCCGTAA TGAACAACAA
2161 ATAATTCCAA ATAGTGCATA ACCAATAAAT ACTTAAAGAT TTGTCTTTCA ACACTAATGT
2221 TTGCATTTCA TGCAATTGTA TTGCTCTGGA AGCACCTTTT AACAGGTTGT ATCATCACAT
2281 GCATTACATT GGAGGATAAG GATATAAGAG ATCATATATA GAATAGAGGA TATGGTGTTT
2341 TAGAAAAAGA AGATGAAGAA TATACATTGT GAAACAGTGC ATGATATAGG CACATCACTT
2401 AATACATTAA AAGCACTGCC AGAAATCTAT GTTGGTGAAC AAATGCTCAC TGGTTGTCTC
2461 ACTTATATCA CCATTCACCA AGTTCACCGT ATGTAACAAG CACTTGGCCC CATGAATTCA
2521 CGCGCCAAGC TTGTAGTGAA CGGTCTGGAC AGAGATTCGT CGCGTCGTCA GCGGAAGGAG
2581 GTGCTTGAAT CCCTGATGCT GCCGGTTCTC TGTGTGTTTT TGGCTTGTGA GATGGGGTTT

2641 AGTCAGTGCA ATTTCAGTGG ATTTTGCTCA ACTCGGTTGG ATTGGCACGA GATTGGTTCC
2701 GCGTGTGACC GTTTGTTGGA GAACATGAAA TCGCGTTGCT CGTTCTGGTT TGGATGCTCT
2761 GTTTTCTGAA CTAGGTTGCT CGAATTGAAT CTGATTGGTT TATGTCTACC TCCGAGTATC
2821 AAGTAAGTTC AGATGAGCGG TTAGCTCGTT TTCAGAGCTA GACGGGATCA CGATCTGGGA
2881 ATACAATTTT TATGAGGCAA ATGCAGCAAA TATAAAGTGA TCTAACTAAT GCCACTTGAT
2941 ATTATGATGA ACATGTTGTT GTGAAACTGG AAAATGCATG CCAAATTGCC AACCATATAC
3001 ATCTCATTAG TAGAGTAACT TCGTGACAGT GAAAGAAAAC TTCATTCTAG CTGAGGGGAG
3061 ATCACATTGA AGGTATTCGT GATATAATAT GTTAGCAAGA TAAGTAGCTA GGATGGTTTA
3121 TTCATAGCAC ATTGGTACCT GGCAGCATGT CAGATAGATA TTTTCTATTT CAGCAATATA
3181 TTGATCTTAC CTTGTGCAAC AGTTTTCTCC ATCCAAGTTC AGTGAAAATC ATTTGTCCTT
3241 GAACTTATCT ACTCAAGCGA TGAACAATAC TCATACACAT GAGCATATGA ATCAGTTCTA
3301 TCTTCTATGA AGCCTTAACC CTAGATTTAT CACAATCTAG TCATCTAGTC CGGCAGACCA
3361 CATTGAACTC CATTGCAATT TGTGAGTTGT TGTATGGCTT CGGAATGGGG TCGAATCTTT
3421 CTTCAATCCA GGATGGAAAT TTTGAGATCT ATACATCACG TTTGGTACAT CTCTACTACT
3481 CTAGTACTAA AAACATGAAG ATTCTGAACA TGGACATTGC ATAGTTCTCC ATCCCAATTC
3541 ACCCTGCAGT GATCGCTGCA CTGGATAATT ATAATATCAG TTAAAATTGA AAATAATGCA
3601 ACTTCATACT TGCATGGTGT CAGTAGTGCC TGCCTAAGAA ATGTGTCTTG TCATAATATG
3661 ATTACATGAA ATATGTTTAC TTCCTCGTTT CTCTTTATTT GTAAGATAAA GAACTAGATA
3721 TGTGGAAAGT AGGATAGCAA AGAGTATGGC CAAACTCTAA TCTTTGCTTT ATTTTTTGGG
3781 ATGGACCCAA AATTTGTTTC TCCTTTACTT CTTTCCCTTT ACAACAATGT TCTTTACTTC
3841 CAATTCTTAT TAACAAAACT CCAAATACAT GCCAAACTGC ATATGTATGT ATGCTATTAA
3901 GGCACATTTA CAAAGCTCCA AGTTTACCTA CTCAATCATT CACATATGGC GATGACTCAA
3961 ACTCTTAATT GTTATCTGTG TAAGCTGTGA CTTGTGTAAC ACATTCTACA AGTCCCATAC

4021 CAATTCTGTT CACAAAAGTT TCTTTGTCCA GCTCATAATT TACAAAACTG CAAAATGCCA
4081 AAGCAATCTG GCACAACCTT ATCATCATAT TTTCTTTCCA CGCATTAAAG CACTGGCAGA
4141 ATTATCTTTG TGTAGATATT CCAAAAGTAT TGGTTGAATA AATGTCCAAA TAAATTCCAT
4201 GCCTCATGAT TTCCAGCTTA TGTGGCCTCC ACTAGGTGGT TTTGCAAAGG CCAAACTCTT
4261 TCCTGGCTTA CACAGCTACC AGCATGTATA AATAGGCCCC TAGGCAACCA TTATTCCATC
4321 ATCCTCAACA ATATTGTCTA CACCATCTGG AATCTTGTTT AACACTAGAA TTGTAGAATC
4381 AGCATCTAGA AGGGCAACCG GGGAAGGTTA AACCCATAGA ACCTCTCCCT TGGATGATGT
4441 AGACCACGCC AGGAATATTT GTGTATCGAG GTACCAAAAG GCCTTGAGGC TGAATGACAC
4501 GTCGGATCAC AAAAGTACCC GTGCACTGGA ATAATTCATT CTTCTCATCG AAGTACTCAG
4561 TCACCCCAGC TTCTGACCTC ACTTTCCGAA GTGGTTCAAA TGCTTGTAGT CTATCAAAAC
4621 TAGAGCATGC AACAATAGCA AGGAGAGCAA ATACGAAAAT GATCTTCATA CTAGACCCAG
4681 ATCTCAGAAG AATGGTCCGT GCCTTAAAAC TTTCCCCAAC CGTGCTAGTT TATGTTGTGA
4741 CTGTCTGCCT CTCTCAGTTT ACTTGGATGC ATTGACAACA TCCTTTTTTG CTATTACTCG
4801 TATTTGCTCT ATAGCTGGTG GCATATCTCA TGTTGAAATT TGCCCTTTTA ATCCAAAATT
4861 GGATGTAATT GAAAGAATCC TACGTGGTAG TTATTTGGAT TTTGGTGTGA AAAAAAATAG
4921 CCTTGTTAGA AGAAGCAAAA TTGGATTTAG TTAAAAGGAT ACTAGATGGT GTTATTTGGA
4981 TTTTGGTGCA AATCAAATTA GGAGGTTGGT TTTATTCAAG TTAAAGTTTG TTTTAAAAAA
5041 ATTCTCCTAA AAAGATAGAT ACTAGATTTG CATATATGCA TTGAAAATTA CATCTTCGCT
5101 TGGCGGTTAT ACTTTTAGTC CCTCTAAATT GTTCAATCAT TTATGATGAA AAGGAAAATC
5161 ATTTTATATC ACAAAGTATT TATGATGAAA GGGGAAAAAT ATTCTGCATG GGTTTGAACA
5221 AAATACGTGG ATTGGTGTAG CCTTAACATA CTTGAAAAGG GTATGATGTT GATGTAGTGC
5281 CCACATGGTG TCGCTTGACA TTAAAACGAT ATGCAGTCAG GATTGAGGAA CATTGCTGAC
5341 AATTTACTAT CGCTGTCTGT GTTGACCACA ATAATTCAGA TGTACCATCC TATCTTCTAA

5401 CTAGAAAGAT GCATGGAAGT TTCTTACATT ATTTCCAGCA CTTGAAATTT TAGTGAAATA
5461 TCATTAAAAC ATAACCACTT ACTTTGCTGT GATATGAAAT AAATGTTTTA TTTCTTGGAA
5521 AGTGGTATAT TCATATATTC TTACAGTAAA TTTATTGATT TTCTTTTCAT TTATTTCTAA
5581 ATTTTAACCA CCCTTTTGGT AGCTTAAGGA AAATTGTATG TTTGACAGTC CTGTTTTCTG
5641 TTGTTTCATC CCTCCAGGAA AACCAGCTAC TAGTATGAAG ATCATTTTCG TATTTGCTCT
5701 CCTTGCTATT GTTGCATGCT CTAGTTTTGA TAGACTACAA GCATTTGAAC CACTTCGGAA
5761 AGTGAGGTCA GAAGCTGGGG TGACTGAGTA CTTCGATGAG AAGAATGAAT TATTCCAGTG
5821 CACGGGTACT TTTGTGATCC GACGTGTCAT TCAGCCTCAA GGCCTTTTGG TACCTCGATA
5881 CACAAATATT CCTGGCGTGG TCTACATCAT CCAAGGGAGA GGTTCTATGG GTTTAACCTT
5941 CCCCGGTTGC CCTTCTAGTG GATCCCCGGG TACCGAGCTC AAACGTTGGT TACATGTACT
6001 CTAGTAATAA GGTGTTGCAT ACTATCGTGT GCAAACACTA GAAATAAGAA CCATTGAATA
6061 AAATATCAAT CATTTTCAGA CTTGCAAATA TTGGGTATTT GGATTTCTGT CCCATGTCCC
6121 TCTTGAAAGC CATGCTGTAC ATGTTGGAGT TCCCCCTTGG ACCCAACCTA CTCCATGCTC
6181 CCATGTTGAT CTTAAATTCC CTGTTCCCCC AGAGCATGTA AATTTTCTTA TGCTAATCAG
6241 AGCAAGCTCG ATGTCTCATT AACATATCCC TATTTGATCA ATGTTGTGGT TGTTTCAAAA
6301 TGTATTGGTG ATAAGTGGTA ACAGTGTACT TCTGGCATAA TGGATAATAA GTGGCGGAGG
6361 TTTGTGAAGA TAAGAGTTGG AAACAGAGCT GCTGTGTATG TTCTAATGAC AGAGAGTTTT
6421 TGGATGGTCA CATAGATCTT GGCCTTTTAC TTTTGATGGA GGGTTGCAGA CGATCATTGA
6481 TGAATGGAGG GCTTTGTTTT TAAGAATCTT ATTAGTGGGT AGTGGTTACT AGTTAGATGT
6541 CTATCAATCT ATAGATAAAA ATAATGTTGT ATATCAAAGG ATTTTTGTTC TTCGCTTTCT
6601 TATGCCCAGT CATTTTCTGA ATTCACGCGT TTTAATTAAC CAATTCGTAA TCATGGTCAT
6661 AGCTGTTTCC TGTGTGAAAT TGTTATCCGC TCACAATTCC ACACAACATA CGAGCCGGAA
6721 GCATAAAGTG TAAAGCCTGG GGTGCCTAAT GAGTGAGCTA ACTCACATTA ATTGCGTTGC

6781 GCTCACTGCC CGCTTTCCAG TCGGGAAACC TGTCGTGCCA GCTGCATTAA TGAATCGGCC
6841 AACGCGCGGG GAGAGGCGGT TTGCGTATTG GAGCTTGAGC TTGGATCAGA TTGTCGTTTC
6901 CCGCCTTCAG TTTAAACTAT CAGTG**TTTGA AAG**

CTB protein coding region is shown in red. RNAi region (RNAi-RAP intron-RNAi) is shown in gray.

A part of RB (Right Border) is indicated by double lines. Mutation is shown in blue.

1	SR**TPQQITDL**	**CAEYHNTQIH**	**TLNDKIFSYT**	**ESLAGKREMA**	**IITFKNGATF**
51	**QVEVPGSQHI**	**DSQKK**AIERM	KDTLR**IAYLT**	**EAK**VEK**LCVW**	**NNKTPHAIAA**
101	**ISMAN**				

Supplementary Figure 7

Identification of CTB (N4Q) protein by shotgun MS/MS in MucoRice-CTB line 19A. Matched peptides are shown in bold red. Protein sequence coverage was 85%.

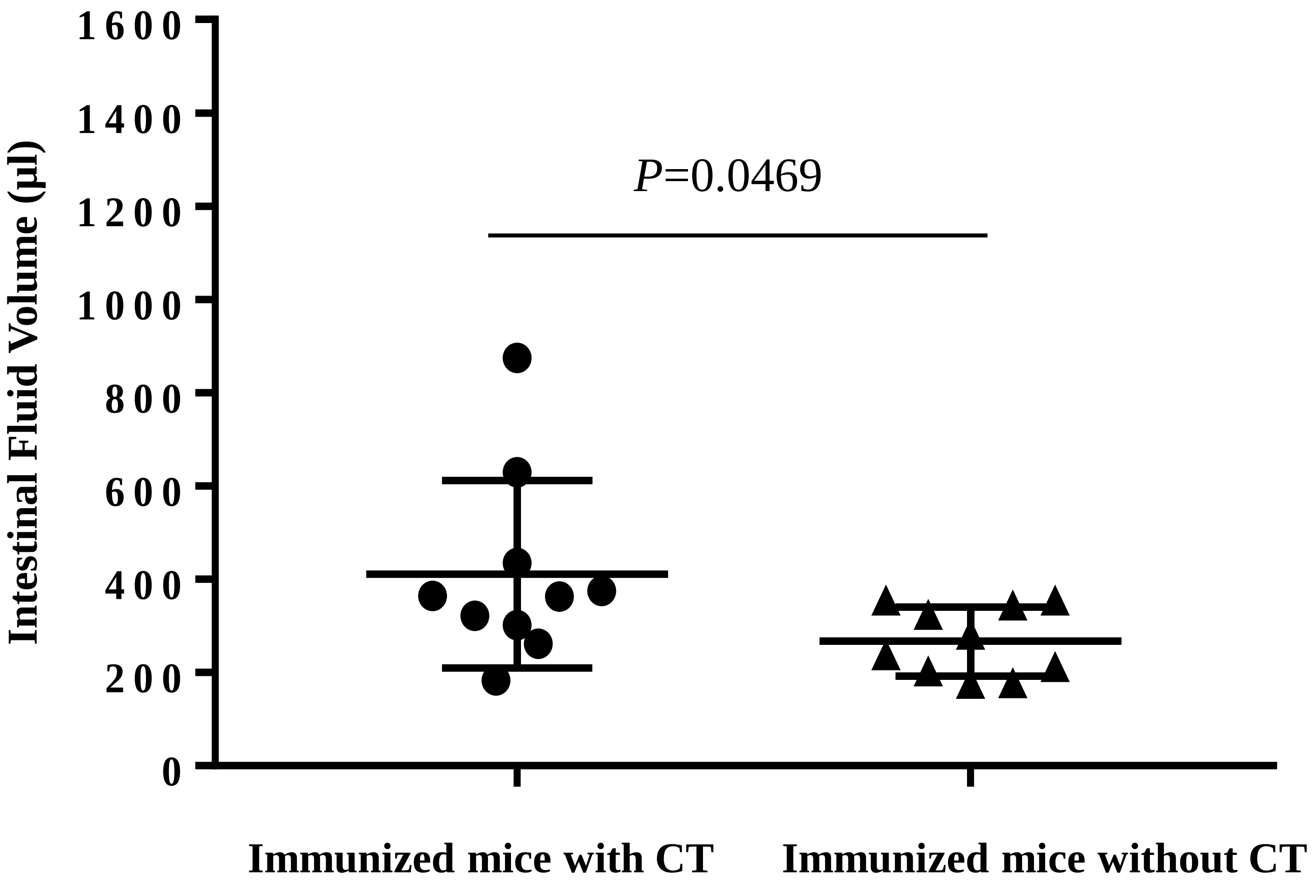

Supplementary Figure 8.

Intestinal fluid volume with (left) and without (right) CT challenge in mice immunized with MucoRice-CTB line 19A.

Group differences in data were evaluated using the two-tailed Student t test.

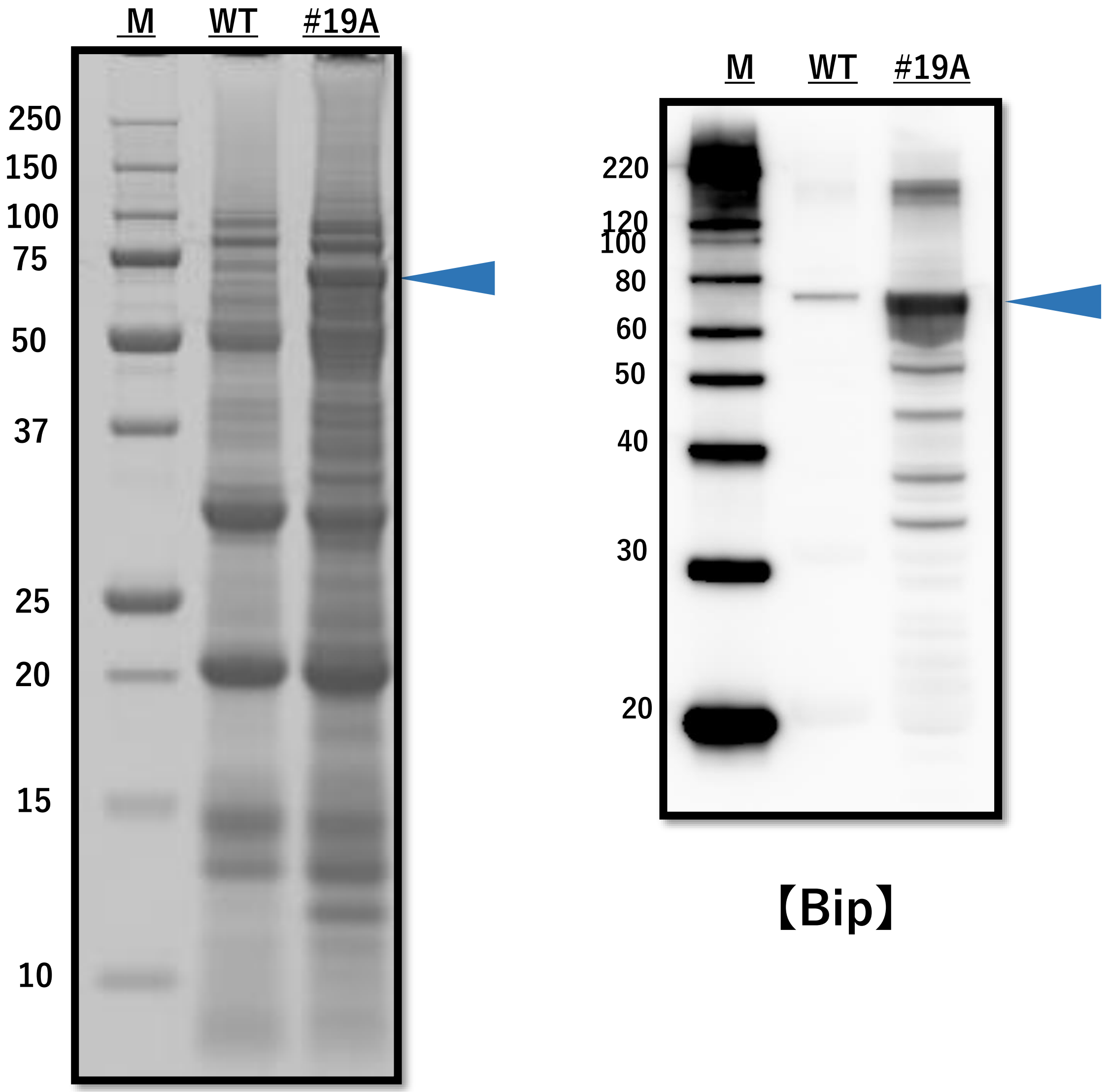

Supplementary Figure 9.
Detection of the heat shock protein Bip in WT and MucoRice-CTB line 19A seed. Total protein was separated by SDS-PAGE (left, Coomassie staining) and analyzed by western blotting (right) with polyclonal antibody raised in rabbits immunized with the recombinant N-terminal domain (residues 26–274) of rice Bip. Endogenous rice Bip was detected as a 75-kDa band in MucoRice-CTB 19A and WT rice (arrowheads), and bands with higher or lower electrophoretic mobility were also found in MucoRice-CTB 19A. M, molecular weight markers (kDa).

Supplementary Table 1. Transformation frequency and HPT marker-free MucoRice-CTB lines

Infection	Calli used for screening (A) *	CTB & HPT genes positive T0 calli (B) **	Ratio (B/A)	CTB protein positive T1 seeds ***	HPT marker–free line
1	53	15	28%	15	#19A
2	36	17	47%	14	none
3	53	15	28%	10	#9B
4	50	12	24%	9	#43B, #50A
Total	192	59	32%	48	4

* Calli used for screening were selected by MS Gelrite medium with hygromycin culture system.
** CTB & HPT genes positive T0 calli were selected by using PCR.
*** CTB protein positive T1 seeds were selected by using SDS-PAGE followed by Western blot analysis with anti CTB Antibody.

Supplementary Table 2. Primer sequence for PCR used in this study

Primer name	DNA sequence	Purpose
CTB-F	5’–ACACCTCAACAGATTACTGA–3’	CTB gene, CTB gene Probe
CTB-R	5’–TCAATTTGCCATACTAATTGCG–3’	CTB gene, CTB gene Probe
HPT-F	5’–TGAACTCACCGCGACGTCT–3’	HPT gene, HPT gene Probe
HPT-R	5’–TCGGCGAGTACTTCTACAC–3’	HPT gene, HPT gene Probe
19A ch1-F	5’– CGATCGAGCCCCTCTCTCTA–3’	#19A Full DNA sequence inserted and RB flanking sequence in chromosome 1
19A ch1-R3	5’– GCAACGCGAGTGACTTGTAC–3’	#19A Full DNA sequence inserted in chromosome 1
RAPint3-R	5’–ACATGAGATATGCCACCAGC–3’	#19A Full DNA sequence inserted in chromosome 1
RAPint4-F	5’–ATGTTGTGACTGTCTGCCTC–3’	#19A Full DNA sequence inserted in chromosome 1
19A ch1-R1	5’–CGAACCTTAATCTGCCCGGT–3’	#19A LB flanking sequence chromosome 1
131out2	5’–CTTTCTTTTGAACTGAACGGG–3’	#19A LB flanking sequence chromosome 1
10TLend5	5’–AGTGGGTAGTGGTTACTAGT–3’	#19A RB flanking sequence chromosome 1
Pro13k-F	5′–CAGGCTGGTAGCGCAACA-3’	Quantitative real-time PCR
Pro13k-R	5′–ACAATCGCCTGAACGCTACT-3’	Quantitative real-time PCR
Glu A-F	5′–ACAAAGAGAAGGATGTGCTTAC-3′	Quantitative real-time PCR
Glu A-R	5′–ATTCTTTATCCGCATTGCCAAC-3’	Quantitative real-time PCR
Glu B-F	5′–CAAGACAAACGCTAACGCCTTC-3′	Quantitative real-time PCR
Glu B-R	5′–TCGATAATCCTGGGTAGTATTG-3′	Quantitative real-time PCR
17SrRNA-F	5′–TTCCGGTCCTATTGTGTTGG-3’	Quantitative real-time PCR
17SrRNA-R	5′–ATGCTTTCGCAGTTGTTCGT-3’	Quantitative real-time PCR

Supplementary Table 3. Environmental parameters for the cultivation of MucoRice-CTB 19A

**Element of Environment**	**Set value**	
	Growing seedlings conditions	Cultivation conditions
**Light / Dark period**	16h / 8h	12h / 12h
**Light intensity**	400 – 500μmol/m^2/s	600– 700μmol/m^2/s
**Temperature**	29C / 24C	27C / 23C
**Relative humidity**	70%	
**CO_2 concentration**	500ppm	
**Nutrient solution**	OAT House Fertilizers #1, #2 and #5	

Supplementary Table 4. DNA sequences to identify reads containing insert-specific sequences

T-DNA vector designed

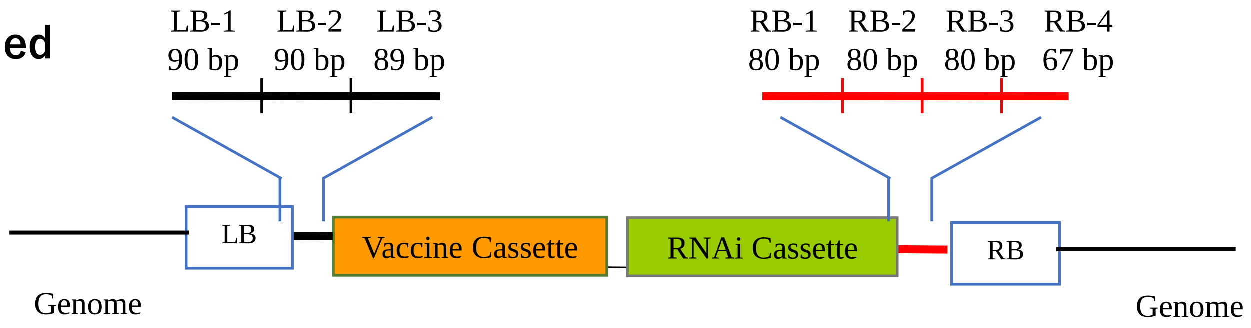

LB-1 (90 bp)	ACAAATTGACGCTTAGACAACTTAATAACACATTGCGGACGTTTTTAATGTACTGAATTAACGCCGAATTGCTCTAGCATTCGCCATTCA
LB-2 (90 bp)	GGCTGCGCAACTGTTGGGAAGGGCGATCGGTGCGGGCCTCTTCGCTATTACGCCAGCTGGCGAAAGGGGGATGTGCTGCAAGGCGATTAA
LB-3 (89 bp)	GTTGGGTAACGCCAGGGTTTTCCCAGTCACGACGTTGTAAAACGACGGCCAGTGCCAAGCTGGCGATCGCTTTGGCGCGCCAAGCTTTT
RB-1 (80 bp)	GAATTCACGCGTTTTAATTAACCAATTCGTAATCATGGTCATAGCTGTTTCCTGTGTGAAATTGTTATCCGCTCACAATT
RB-2 (80 bp)	CCACACAACATACGAGCCGGAAGCATAAAGTGTAAAGCCTGGGGTGCCTAATGAGTGAGCTAACTCACATTAATTGCGTT
RB-3 (80 bp)	GCGCTCACTGCCCGCTTTCCAGTCGGGAAACCTGTCGTGCCAGCTGCATTAATGAATCGGCCAACGCGCGGGGAGAGGCG
RB-4 (67 bp)	GTTTGCGTATTGGAGCTTGAGCTTGGATCAGATTGTCGTTTCCCGCCTTCAGTTTAAACTATCAGTG

Sapplementary Table 5. Salt-soluble proteins of MucoRice-CTB19A and WT rice identified in both rices by Shot-gun MS analysis

		19A				WT				
Accession	Description	# PSMs	# AAs	MW [kDa]	calc. pI	# PSMs	# AAs	MW [kDa]	calc. pI	#PSMs 19A/WT
XP_015625618.1	heat shock 70 kDa protein BIP1 [Oryza sativa Japonica Group]	565	665	73.3	5.19	15	665	73.3	5.19	37.667
EEC75043.1	hypothetical protein OsI_11143 [Oryza sativa Indica Group]	15	256	28.5	6.04	1	256	28.5	6.04	15.000
XP_015627045.1	protein disulfide isomerase-like 1-4 [Oryza sativa Japonica Group]	23	563	62.2	4.93	2	563	62.2	4.93	11.500
XP_015611801.1	protein disulfide isomerase-like 2-3 [Oryza sativa Japonica Group]	34	441	47.3	5.58	4	441	47.3	5.58	8.500
EAY91599.1	hypothetical protein OsI_13234 [Oryza sativa Indica Group]	24	426	47.0	5.21	3	426	47.0	5.21	8.000
XP_015613091.1	lysosomal Pro-X carboxypeptidase [Oryza sativa Japonica Group]	14	517	57.4	5.31	2	517	57.4	5.31	7.000
BAS73554.1	Os01g0663400, partial [Oryza sativa Japonica Group]	44	445	47.7	5.96	7	445	47.7	5.96	6.286
BAD38226.1	putative formate--tetrahydrofolate ligase [Oryza sativa Japonica Group]	6	639	68.1	7.01	1	639	68.1	7.01	6.000
EEE60487.1	hypothetical protein OsJ_13773 [Oryza sativa Japonica Group]	37	895	99.9	5.95	7	895	99.9	5.95	5.286
ABR25447.1	40S ribosomal protein S14, partial [Oryza sativa Indica Group]	5	145	15.7	10.20	1	145	15.7	10.20	5.000
BAD88157.1	protein kinase-like [Oryza sativa Japonica Group]	5	463	49.6	7.97	1	463	49.6	7.97	5.000
XP_015651405.1	peptidyl-prolyl cis-trans isomerase FKBP15-1 [Oryza sativa Japonica Group]	5	154	16.3	5.38	1	154	16.3	5.38	5.000
XP_015618966.1	probable mediator of RNA polymerase II transcription subunit 37c [Oryza sativa Japonica Group]	74	648	70.9	5.21	15	648	70.9	5.21	4.933
BAA06876.1	aspartic protease [Oryza sativa]	28	509	54.1	5.26	6	509	54.1	5.26	4.667
EEC79860.1	hypothetical protein OsI_21345 [Oryza sativa Indica Group]	8	475	52.4	8.00	2	475	52.4	8.00	4.000
XP_015611898.1	polyadenylate-binding protein 2 [Oryza sativa Japonica Group]	4	662	71.6	6.83	1	662	71.6	6.83	4.000
XP_015647977.1	MD-2-related lipid-recognition protein ROSY1 [Oryza sativa Japonica Group]	4	152	16.2	5.50	1	152	16.2	5.50	4.000
ACJ54890.1	heat shock protein 70 [Oryza sativa Japonica Group]	75	653	71.6	5.43	19	653	71.6	5.43	3.947
AAT47049.1	unknown protein [Oryza sativa Japonica Group]	3	109	11.7	5.36	1	109	11.7	5.36	3.000
BAA77337.1	Nad-dependent formate dehydrogenase [Oryza sativa]	6	376	41.2	7.34	2	376	41.2	7.34	3.000
BAD68706.1	putative ketol-acid reductoisomerase precursor [Oryza sativa Japonica Group]	9	548	59.6	6.07	3	548	59.6	6.07	3.000
BAH92448.1	Os03g0854400 [Oryza sativa Japonica Group]	3	176	17.9	10.14	1	176	17.9	10.14	3.000
BAS99345.1	Os06g0701100, partial [Oryza sativa Japonica Group]	3	400	45.6	5.67	1	400	45.6	5.67	3.000
BAT15366.1	Os11g0701100, partial [Oryza sativa Japonica Group]	30	284	31.0	6.54	10	284	31.0	6.54	3.000
EEC77777.1	hypothetical protein OsI_16938 [Oryza sativa Indica Group]	3	168	18.5	7.81	1	168	18.5	7.81	3.000
EEE68955.1	hypothetical protein OsJ_27847 [Oryza sativa Japonica Group]	6	236	24.1	8.59	2	236	24.1	8.59	3.000
XP_015618089.1	probable aspartyl aminopeptidase [Oryza sativa Japonica Group]	3	478	52.4	6.92	1	478	52.4	6.92	3.000
XP_015622112.1	60S ribosomal protein L30 [Oryza sativa Japonica Group]	3	111	12.4	9.58	1	111	12.4	9.58	3.000
XP_015640215.1	serine carboxypeptidase 2 isoform X2 [Oryza sativa Japonica Group]	11	443	49.2	6.60	4	443	49.2	6.60	2.750
XP_015650060.1	60S acidic ribosomal protein P1 [Oryza sativa Japonica Group]	11	110	11.1	4.51	4	110	11.1	4.51	2.750
3FR7_A	Chain A, Ketol-Acid Reductoisomerase (Kari) In Complex With Mg2+	10	525	57.3	5.67	4	525	57.3	5.67	2.500
XP_015622490.1	60S ribosomal protein L23A [Oryza sativa Japonica Group]	5	152	17.0	10.30	2	152	17.0	10.30	2.500
XP_015627232.1	60S ribosomal protein L12-1 [Oryza sativa Japonica Group]	5	166	17.7	9.16	2	166	17.7	9.16	2.500
EEE62108.1	hypothetical protein OsJ_16892 [Oryza sativa Japonica Group]	14	359	38.2	6.16	6	359	38.2	6.16	2.333
XP_015647572.1	thioredoxin H1 [Oryza sativa Japonica Group]	14	122	13.1	5.30	6	122	13.1	5.30	2.333
XP_015614147.1	nucleoside diphosphate kinase 1 [Oryza sativa Japonica Group]	18	151	16.8	7.50	8	151	16.8	7.50	2.250
BAF14618.2	Os04g0404400 [Oryza sativa Japonica Group]	29	295	31.2	5.02	13	295	31.2	5.02	2.231
EEC81650.1	hypothetical protein OsI_25188 [Oryza sativa Indica Group]	17	533	57.1	6.35	8	533	57.1	6.35	2.125
AAA33918.1	UDP-glucose starch glycosyl transferase, partial [Oryza sativa]	4	191	22.0	5.88	2	191	22.0	5.88	2.000
AAL40390.1	C13 cysteine proteinase precursor [Oryza sativa Indica Group]	2	465	51.9	6.04	1	465	51.9	6.04	2.000
AAO23563.1	aspartate aminotransferase, partial [Oryza sativa]	2	414	45.8	6.28	1	414	45.8	6.28	2.000
BAA02157.1	40S subunit ribosomal protein [Oryza sativa Japonica Group]	2	117	13.0	9.63	1	117	13.0	9.63	2.000
BAA94761.1	adenylate kinase, partial [Oryza sativa]	4	209	22.8	8.06	2	209	22.8	8.06	2.000
BAD04054.1	heat shock protein 90 [Oryza sativa Japonica Group]	6	699	80.2	5.06	3	699	80.2	5.06	2.000
BAF17027.1	Os05g0295800 [Oryza sativa Japonica Group]	4	189	21.3	6.23	2	189	21.3	6.23	2.000

BAF20971.1	Os07g0184300 [Oryza sativa Japonica Group]	2	118	13.4	10.73	1	118	13.4	10.73	2.000
BAT05491.1	Os08g0424500, partial [Oryza sativa Japonica Group]	2	389	42.3	5.24	1	389	42.3	5.24	2.000
BAT16466.1	Os12g0235800, partial [Oryza sativa Japonica Group]	4	350	38.9	6.21	2	350	38.9	6.21	2.000
EAY85029.1	hypothetical protein OsI_06386 [Oryza sativa Indica Group]	4	878	98.3	5.17	2	878	98.3	5.17	2.000
EEC71703.1	hypothetical protein OsI_04213 [Oryza sativa Indica Group]	6	559	60.7	5.60	3	559	60.7	5.60	2.000
NP_001147283.1	40S ribosomal protein S28 [Zea mays]	2	65	7.5	11.17	1	65	7.5	11.17	2.000
XP_015611712.1	glutathione transferase GST 23 [Oryza sativa Japonica Group]	2	223	25.2	5.71	1	223	25.2	5.71	2.000
XP_015622168.1	nascent polypeptide-associated complex subunit alpha-like protein 1 [Oryza sativa Japonica Group]	6	202	22.1	4.55	3	202	22.1	4.55	2.000
XP_015627578.1	60S ribosomal protein L6-3 [Oryza sativa Japonica Group]	2	219	24.2	10.11	1	219	24.2	10.11	2.000
XP_015628386.1	cysteine proteinase inhibitor 8 [Oryza sativa Japonica Group]	6	123	12.9	8.69	3	123	12.9	8.69	2.000
XP_015633089.1	peptide methionine sulfoxide reductase B5 [Oryza sativa Japonica Group]	2	136	14.7	7.24	1	136	14.7	7.24	2.000
XP_015633778.1	2-oxoglutarate-dependent dioxygenase DAO [Oryza sativa Japonica Group]	2	300	32.1	5.82	1	300	32.1	5.82	2.000
XP_015642917.1	GDSL esterase/lipase At4g01130 [Oryza sativa Japonica Group]	4	379	40.9	8.15	2	379	40.9	8.15	2.000
XP_015647517.1	uncharacterized protein LOC4343118 isoform X2 [Oryza sativa Japonica Group]	2	543	58.4	6.06	1	543	58.4	6.06	2.000
XP_015650330.1	mitochondrial import inner membrane translocase subunit TIM8 [Oryza sativa Japonica Group]	2	73	8.3	5.31	1	73	8.3	5.31	2.000
XP_025878742.1	leucine aminopeptidase 2, chloroplastic [Oryza sativa Japonica Group]	2	532	55.1	5.80	1	532	55.1	5.80	2.000
XP_025879244.1	non-specific lipid-transfer protein 4 [Oryza sativa Japonica Group]	2	81	8.2	7.96	1	81	8.2	7.96	2.000
AAA82047.1	glyceraldehyde-3-phosphate dehydrogenase [Oryza sativa Indica Group]	19	337	36.5	7.11	10	337	36.5	7.11	1.900
AAN05517.1	unknown protein [Oryza sativa Japonica Group]	11	317	35.4	5.87	6	317	35.4	5.87	1.833
XP_015625382.1	glyceraldehyde-3-phosphate dehydrogenase 3, cytosolic [Oryza sativa Japonica Group]	23	337	36.5	7.88	13	337	36.5	7.88	1.769
AAX95683.1	hypothetical protein [Oryza sativa Japonica Group]	5	103	11.6	5.90	3	103	11.6	5.90	1.667
BAF23792.1	Os08g0434300, partial [Oryza sativa Japonica Group]	5	356	37.4	6.70	3	356	37.4	6.70	1.667
BAT14238.1	Os11g0525600, partial [Oryza sativa Japonica Group]	5	904	101.4	6.24	3	904	101.4	6.24	1.667
EAZ00429.1	hypothetical protein OsI_22453 [Oryza sativa Indica Group]	5	413	43.1	4.77	3	413	43.1	4.77	1.667
XP_015614294.1	malate dehydrogenase, cytoplasmic [Oryza sativa Japonica Group]	29	332	35.5	6.09	18	332	35.5	6.09	1.611
AAA57130.1	manganese superoxide dismutase [Oryza sativa]	18	231	24.9	7.02	12	231	24.9	7.02	1.500
AAN05528.1	mitochondrial chaperonin-60 [Oryza sativa Japonica Group]	3	574	60.8	5.87	2	574	60.8	5.87	1.500
ABG00012.1	RuBisCO subunit binding-protein alpha subunit, chloroplast precursor, putative, expressed [Oryza sativa Japonica Group]	3	479	50.4	5.08	2	479	50.4	5.08	1.500
ABR25841.1	40S ribosomal protein S8, partial [Oryza sativa Indica Group]	3	168	18.8	9.69	2	168	18.8	9.69	1.500
CAD41669.3	OSJNBa0019K04.16 [Oryza sativa Japonica Group]	6	431	44.5	6.65	4	431	44.5	6.65	1.500
XP_015615329.1	adenylate kinase 4 [Oryza sativa Japonica Group]	3	243	26.7	7.80	2	243	26.7	7.80	1.500
XP_015632687.1	subtilisin-like protease SBT1.7 [Oryza sativa Japonica Group]	3	764	78.5	6.67	2	764	78.5	6.67	1.500
XP_015632967.1	uncharacterized protein At5g02240 [Oryza sativa Japonica Group]	3	257	27.9	6.81	2	257	27.9	6.81	1.500
XP_015648168.1	acyl-CoA-binding domain-containing protein 1-like [Oryza sativa Japonica Group]	3	91	10.1	5.22	2	91	10.1	5.22	1.500
BAF11185.1	Os03g0197300 [Oryza sativa Japonica Group]	29	601	68.2	5.71	21	601	68.2	5.71	1.381
ABD57308.1	UDP-glucose pyrophosphorylase [Oryza sativa Indica Group]	26	469	51.6	5.59	19	469	51.6	5.59	1.368
A6N0M9.1	RecName: Full=Nucleoside diphosphate kinase 1; AltName: Full=Nucleoside diphosphate kinase I; Short=NDK I; Short=NDP kinase I; Short=NDPK I	12	149	16.8	6.80	9	149	16.8	6.80	1.333
EAY90376.1	hypothetical protein OsI_11954 [Oryza sativa Indica Group]	4	310	33.4	5.08	3	310	33.4	5.08	1.333
Q75H81.1	RecName: Full=Serpin-ZXA; AltName: Full=OrysaZxa	4	396	42.1	6.15	3	396	42.1	6.15	1.333
XP_015645300.1	1-Cys peroxiredoxin A [Oryza sativa Japonica Group]	41	220	24.0	6.43	31	220	24.0	6.43	1.323
AGT59174.1	glutelin, partial [Oryza sativa Indica Group]	5	454	51.2	8.68	4	454	51.2	8.68	1.250
Q65XA0.1	RecName: Full=Probable glutathione S-transferase DHAR1, cytosolic; AltName: Full=GSH-dependent dehydroascorbate reductase 1	15	213	23.6	6.21	12	213	23.6	6.21	1.250
XP_015639252.1	fructose-bisphosphate aldolase 1, cytoplasmic [Oryza sativa Japonica Group]	21	358	38.8	7.33	17	358	38.8	7.33	1.235
ADM86861.1	triosephosphate isomerase [Oryza sativa Japonica Group]	24	253	27.0	5.49	20	253	27.0	5.49	1.200
BAA03711.1	brain specific protein [Oryza sativa]	6	260	29.1	4.83	5	260	29.1	4.83	1.200
EAY89381.1	hypothetical protein OsI_10886 [Oryza sativa Indica Group]	6	159	17.7	6.61	5	159	17.7	6.61	1.200
XP_015621604.1	malate dehydrogenase, mitochondrial [Oryza sativa Japonica Group]	12	340	35.4	8.56	10	340	35.4	8.56	1.200
NP_001105455.2	calmodulin [Zea mays]	7	149	16.8	4.27	6	149	16.8	4.27	1.167
XP_015620921.1	guanine nucleotide-binding protein subunit beta-like protein A [Oryza sativa Japonica Group]	7	334	36.2	6.44	6	334	36.2	6.44	1.167
XP_015645043.1	reactive Intermediate Deaminase A, chloroplastic [Oryza sativa Japonica Group]	7	180	18.8	9.39	6	180	18.8	9.39	1.167

XP_015639465.1	malate dehydrogenase, mitochondrial [Oryza sativa Japonica Group]	11	340	35.4	8.10	10	340	35.4	8.10	1.100
XP_015628337.1	cupincin [Oryza sativa Japonica Group]	83	470	52.1	7.25	77	470	52.1	7.25	1.078
XP_015629108.1	late embryogenesis abundant protein 17 [Oryza sativa Japonica Group]	16	344	36.8	6.83	15	344	36.8	6.83	1.067
XP_015645679.1	17kDa alpha-amylase/trypsin inhibitor 1 [Oryza sativa Japonica Group]	101	158	16.4	7.50	95	158	16.4	7.50	1.063
AAA33917.1	superoxide dismutase [Oryza sativa Japonica Group]	1	152	15.2	6.18	1	152	15.2	6.18	1.000
AAA70046.1	lipid transfer protein precursor, partial [Oryza sativa]	2	99	10.0	8.51	2	99	10.0	8.51	1.000
AAF73828.1	aldehyde dehydrogenase [Oryza sativa]	1	549	59.3	6.80	1	549	59.3	6.80	1.000
AAG28777.1	citrate synthase [Oryza sativa]	1	472	52.2	7.88	1	472	52.2	7.88	1.000
ABA92030.1	Alcohol dehydrogenase 1, putative, expressed [Oryza sativa Japonica Group]	10	345	37.5	6.65	10	345	37.5	6.65	1.000
ABF97261.1	40S ribosomal protein S3, putative, expressed [Oryza sativa Japonica Group]	1	205	22.8	9.54	1	205	22.8	9.54	1.000
ABF99925.1	NAC domain containing protein, expressed [Oryza sativa Japonica Group]	2	146	16.1	6.01	2	146	16.1	6.01	1.000
ABR25518.1	60S ribosomal protein l22-2, partial [Oryza sativa Indica Group]	2	88	10.2	9.61	2	88	10.2	9.61	1.000
ABR25651.1	regulator of ribonuclease activity a, partial [Oryza sativa Indica Group]	1	167	17.9	5.88	1	167	17.9	5.88	1.000
ABR26039.1	cbs domain protein, partial [Oryza sativa Indica Group]	2	141	15.6	8.15	2	141	15.6	8.15	1.000
ABR26121.1	salt stress-induced protein, partial [Oryza sativa Indica Group]	1	104	11.0	4.70	1	104	11.0	4.70	1.000
BAA78385.1	heat shock protein 26 [Oryza sativa]	1	239	26.6	6.83	1	239	26.6	6.83	1.000
BAC06273.1	putative 60S ribosomal protein L5 [Oryza sativa Japonica Group]	7	301	34.3	9.09	7	301	34.3	9.09	1.000
BAD81175.1	putative cysteine proteinase inhibitor [Oryza sativa Japonica Group]	2	208	23.3	6.23	2	208	23.3	6.23	1.000
BAS75388.1	Os01g0866600, partial [Oryza sativa Japonica Group]	1	73	7.8	6.77	1	73	7.8	6.77	1.000
BAS81339.1	Os02g0793700, partial [Oryza sativa Japonica Group]	1	101	10.7	6.74	1	101	10.7	6.74	1.000
BAS81910.1	Os03g0109600, partial [Oryza sativa Japonica Group]	2	100	11.1	9.38	2	100	11.1	9.38	1.000
BAT09494.1	Os09g0568900, partial [Oryza sativa Japonica Group]	2	82	8.4	9.04	2	82	8.4	9.04	1.000
EAY97222.1	hypothetical protein OsI_19141 [Oryza sativa Indica Group]	1	169	16.0	6.89	1	169	16.0	6.89	1.000
EAZ00738.1	hypothetical protein OsI_22765 [Oryza sativa Indica Group]	1	81	8.7	6.32	1	81	8.7	6.32	1.000
EAZ01248.1	hypothetical protein OsI_23273 [Oryza sativa Indica Group]	1	164	17.6	5.80	1	164	17.6	5.80	1.000
EAZ12194.1	hypothetical protein OsJ_02079 [Oryza sativa Japonica Group]	2	243	26.1	5.57	2	243	26.1	5.57	1.000
EEC77750.1	hypothetical protein OsI_16870 [Oryza sativa Indica Group]	1	140	14.4	7.74	1	140	14.4	7.74	1.000
EEC78570.1	hypothetical protein OsI_18555 [Oryza sativa Indica Group]	5	502	52.7	7.75	5	502	52.7	7.75	1.000
EEC83682.1	hypothetical protein OsI_29480 [Oryza sativa Indica Group]	2	226	23.3	8.10	2	226	23.3	8.10	1.000
EEE54097.1	hypothetical protein OsJ_00845 [Oryza sativa Japonica Group]	1	161	17.3	7.50	1	161	17.3	7.50	1.000
XP_006659067.1	PREDICTED: probable calcium-binding protein CML7 [Oryza brachyantha]	1	148	16.7	5.06	1	148	16.7	5.06	1.000
XP_015611390.1	uncharacterized protein LOC4346597 [Oryza sativa Japonica Group]	1	185	17.3	7.65	1	185	17.3	7.65	1.000
XP_015613005.1	uncharacterized protein LOC4348196 [Oryza sativa Japonica Group]	1	386	42.6	5.02	1	386	42.6	5.02	1.000
XP_015617060.1	translationally-controlled tumor protein homolog [Oryza sativa Japonica Group]	1	168	18.9	4.68	1	168	18.9	4.68	1.000
XP_015617463.1	uncharacterized protein LOC4327239 [Oryza sativa Japonica Group]	4	432	46.4	6.44	4	432	46.4	6.44	1.000
XP_015619738.1	osmotin-like protein [Oryza sativa Japonica Group]	1	233	23.4	8.02	1	233	23.4	8.02	1.000
XP_015621051.1	cysteine proteinase inhibitor 5 [Oryza sativa Japonica Group]	1	148	15.7	6.13	1	148	15.7	6.13	1.000
XP_015622003.1	peroxiredoxin-2C [Oryza sativa Japonica Group]	5	162	17.3	5.88	5	162	17.3	5.88	1.000
XP_015622836.1	outer envelope membrane protein 7 [Oryza sativa Japonica Group]	1	101	10.4	4.55	1	101	10.4	4.55	1.000
XP_015627405.1	2-Cys peroxiredoxin BAS1, chloroplastic [Oryza sativa Japonica Group]	4	261	28.1	6.00	4	261	28.1	6.00	1.000
XP_015628927.1	793	2	114	12.7	7.81	2	114	12.7	7.81	1.000
XP_015629015.1	PLASMODESMATA CALLOSE-BINDING PROTEIN 2 [Oryza sativa Japonica Group]	2	175	17.8	4.83	2	175	17.8	4.83	1.000
XP_015630351.1	proteasome subunit alpha type-6 isoform X2 [Oryza sativa Japonica Group]	2	246	27.6	6.65	2	246	27.6	6.65	1.000
XP_015632320.1	uncharacterized protein LOC4334013 [Oryza sativa Japonica Group]	1	82	8.6	5.15	1	82	8.6	5.15	1.000
XP_015632604.1	60S ribosomal protein L19-1 [Oryza sativa Japonica Group]	1	208	24.1	11.39	1	208	24.1	11.39	1.000
XP_015635492.1	probable inactive UDP-arabinopyranose mutase 2 [Oryza sativa Japonica Group]	1	347	38.9	6.44	1	347	38.9	6.44	1.000
XP_015636840.1	dihydrolipoyllysine-residue succinyltransferase component of 2-oxoglutarate dehydrogenase complex 1, mitochondrial isoform X2 [Oryza sativa Japonica Group]	1	439	48.1	8.38	1	439	48.1	8.38	1.000
XP_015637356.1	uncharacterized protein LOC4338014 [Oryza sativa Japonica Group]	1	89	9.1	8.40	1	89	9.1	8.40	1.000
XP_015640756.1	photosynthetic NDH subunit of lumenal location 5, chloroplastic [Oryza sativa Japonica Group]	1	250	26.6	9.29	1	250	26.6	9.29	1.000
XP_015643120.1	probable alpha-glucosidase Os06g0675700 isoform X3 [Oryza sativa Japonica Group]	27	885	96.3	8.59	27	885	96.3	8.59	1.000

XP_015650677.1	acyl carrier protein 3, chloroplastic [Oryza sativa Japonica Group]	1	138	14.5	5.12	1	138	14.5	5.12	1.000
XP_025883358.1	copper transport protein ATX1 isoform X2 [Oryza sativa Japonica Group]	1	79	8.3	7.96	1	79	8.3	7.96	1.000
AAA72362.1	unnamed protein product [Oryza sativa Japonica Group]	314	174	19.8	6.96	317	174	19.8	6.96	0.991
XP_015613809.1	fructose-bisphosphate aldolase 3, cytoplasmic [Oryza sativa Japonica Group]	35	358	38.8	8.16	37	358	38.8	8.16	0.946
XP_015629251.1	oleosin 18 kDa [Oryza sativa Japonica Group]	9	172	17.2	10.10	10	172	17.2	10.10	0.900
EAZ04671.1	hypothetical protein OsI_26825 [Oryza sativa Indica Group]	22	234	23.7	6.20	25	234	23.7	6.20	0.880
CAA40604.1	rab25 product [Oryza sativa Japonica Group]	6	228	24.7	10.45	7	228	24.7	10.45	0.857
EAZ38239.1	hypothetical protein OsJ_22614 [Oryza sativa Japonica Group]	6	204	21.7	9.26	7	204	21.7	9.26	0.857
EEC74867.1	hypothetical protein OsI_10758 [Oryza sativa Indica Group]	11	445	48.0	5.47	13	445	48.0	5.47	0.846
BAD35232.1	putative chaperonin 21 precursor [Oryza sativa Japonica Group]	5	216	23.1	6.00	6	216	23.1	6.00	0.833
Q75GX9.1	RecName: Full=63 kDa globulin-like protein; AltName: Allergen=Ory s GLP63; Flags: Precursor	269	562	63.4	8.13	326	562	63.4	8.13	0.825
P37833.1	RecName: Full=Aspartate aminotransferase, cytoplasmic; AltName: Full=Transaminase A	4	407	44.5	7.94	5	407	44.5	7.94	0.800
XP_015625227.1	peptidyl-prolyl cis-trans isomerase [Oryza sativa Japonica Group]	7	172	18.3	8.34	9	172	18.3	8.34	0.778
XP_015649739.1	glycine-rich protein 2 [Oryza sativa Japonica Group]	7	197	18.7	6.76	9	197	18.7	6.76	0.778
XP_015644193.1	phosphoglycerate kinase, cytosolic [Oryza sativa Japonica Group]	16	401	42.3	6.61	21	401	42.3	6.61	0.762
AAK16176.1	translation initiation factor 5A [Oryza sativa Japonica Group]	3	161	17.5	6.14	4	161	17.5	6.14	0.750
EEE64042.1	hypothetical protein OsJ_18871 [Oryza sativa Japonica Group]	3	121	13.3	6.07	4	121	13.3	6.07	0.750
XP_015640796.1	thioredoxin H-type [Oryza sativa Japonica Group]	3	121	13.2	5.08	4	121	13.2	5.08	0.750
XP_015650763.1	fasciclin-like arabinogalactan protein 1 [Oryza sativa Japonica Group]	6	415	43.6	8.24	8	415	43.6	8.24	0.750
Q01881.2	RecName: Full=Seed allergenic protein RA5; AltName: Allergen=Ory s aA_TI; Flags: Precursor	23	160	17.3	8.03	32	160	17.3	8.03	0.719
AAK55782.1	Hypothetical protein [Oryza sativa]	10	230	23.3	6.33	14	230	23.3	6.33	0.714
XP_015614438.1	alanine aminotransferase 2 [Oryza sativa Japonica Group]	7	483	52.6	6.65	10	483	52.6	6.65	0.700
XP_015631876.1	pyruvate decarboxylase 2 [Oryza sativa Japonica Group]	7	605	64.7	5.81	10	605	64.7	5.81	0.700
EAZ06557.1	hypothetical protein OsI_28805 [Oryza sativa Indica Group]	9	150	15.2	8.51	13	150	15.2	8.51	0.692
ABI74567.1	phosphoglycerate kinase [Oryza sativa Indica Group]	11	402	42.1	5.86	16	402	42.1	5.86	0.688
XP_015629184.1	uncharacterized protein LOC4332421 [Oryza sativa Japonica Group]	11	139	15.0	5.86	16	139	15.0	5.86	0.688
XP_015632814.1	vicilin-like seed storage protein At2g28490 [Oryza sativa Japonica Group]	19	565	61.4	7.53	28	565	61.4	7.53	0.679
BAB56043.1	lipoprotein-like [Oryza sativa Japonica Group]	2	252	28.0	7.93	3	252	28.0	7.93	0.667
BAF29005.1	Os12g0115000, partial [Oryza sativa Japonica Group]	4	110	10.7	8.34	6	110	10.7	8.34	0.667
BAS94548.1	Os05g0477900, partial [Oryza sativa Japonica Group]	2	83	8.1	9.10	3	83	8.1	9.10	0.667
EAY75782.1	hypothetical protein OsI_03698 [Oryza sativa Indica Group]	4	573	63.2	7.44	6	573	63.2	7.44	0.667
XP_015612407.1	cytosolic isocitrate dehydrogenase [NADP] [Oryza sativa Japonica Group]	2	412	46.0	6.80	3	412	46.0	6.80	0.667
XP_015613274.1	low-temperature-induced 65 kDa protein [Oryza sativa Japonica Group]	12	446	45.6	4.87	18	446	45.6	4.87	0.667
XP_015643985.1	late embryogenesis abundant protein D-34 [Oryza sativa Japonica Group]	8	277	27.9	4.36	12	277	27.9	4.36	0.667
EEC81520.1	hypothetical protein OsI_24909 [Oryza sativa Indica Group]	5	138	14.8	5.52	8	138	14.8	5.52	0.625
XP_015626227.1	14-3-3-like protein GF14-E isoform X2 [Oryza sativa Japonica Group]	5	262	29.7	4.77	8	262	29.7	4.77	0.625
XP_015623194.1	poly [ADP-ribose] polymerase 3 isoform X8 [Oryza sativa Japonica Group]	8	831	92.3	5.63	13	831	92.3	5.63	0.615
XP_015638049.1	embryonic abundant protein 1 [Oryza sativa Japonica Group]	8	95	10.2	5.60	13	95	10.2	5.60	0.615
XP_015645309.1	17kDa alpha-amylase/trypsin inhibitor 2 [Oryza sativa Japonica Group]	120	159	16.5	7.50	198	159	16.5	7.50	0.606
AIC77182.1	lipid transfer protein 2 [Oryza sativa]	3	95	9.4	8.95	5	95	9.4	8.95	0.600
XP_015612568.1	uncharacterized protein LOC4346387 [Oryza sativa Japonica Group]	3	75	7.8	5.86	5	75	7.8	5.86	0.600
XP_015617770.1	uncharacterized protein DDB_G0286299 [Oryza sativa Japonica Group]	3	331	35.3	4.98	5	331	35.3	4.98	0.600
XP_015622173.1	ubiquitin-fold modifier 1 [Oryza sativa Japonica Group]	3	102	10.4	9.61	5	102	10.4	9.61	0.600
XP_015625436.1	embryonic protein DC-8 [Oryza sativa Japonica Group]	30	471	47.3	6.87	51	471	47.3	6.87	0.588
P55142.2	RecName: Full=Glutaredoxin-C6; AltName: Full=Glutaredoxin-C2 homolog 1	5	112	11.8	6.04	9	112	11.8	6.04	0.556
Q6YZX6.1	RecName: Full=Putative aconitate hydratase, cytoplasmic; Short=Aconitase; AltName: Full=Citrate hydro-lyase	5	898	98.0	6.01	9	898	98.0	6.01	0.556
ABR25697.1	polyubiquitin containing 7 ubiquitin monomers, partial [Oryza sativa Indica Group]	6	201	22.6	8.28	12	201	22.6	8.28	0.500
BAD09607.1	putative superoxide dismutase [Cu-Zn], chloroplast precursor [Oryza sativa Japonica Group]	1	203	20.5	6.25	2	203	20.5	6.25	0.500
BAD09646.1	putative lipid transfer protein precursor [Oryza sativa Japonica Group]	1	120	12.0	7.84	2	120	12.0	7.84	0.500
BAS71712.1	Os01g0300200, partial [Oryza sativa Japonica Group]	1	487	52.9	8.10	2	487	52.9	8.10	0.500

BAS75922.1	Os01g0919900, partial [Oryza sativa Japonica Group]	1	283	32.3	5.92	2	283	32.3	5.92	0.500
EAZ04317.1	hypothetical protein OsI_26458 [Oryza sativa Indica Group]	2	335	34.0	8.12	4	335	34.0	8.12	0.500
EAZ09241.1	hypothetical protein OsI_31514 [Oryza sativa Indica Group]	2	245	25.5	6.27	4	245	25.5	6.27	0.500
EAZ31805.1	hypothetical protein OsJ_15961 [Oryza sativa Japonica Group]	1	116	12.9	11.27	2	116	12.9	11.27	0.500
EAZ45481.1	hypothetical protein OsJ_30136 [Oryza sativa Japonica Group]	1	245	26.0	8.53	2	245	26.0	8.53	0.500
EEC83981.1	hypothetical protein OsI_30128 [Oryza sativa Indica Group]	2	361	38.5	6.39	4	361	38.5	6.39	0.500
EEE60187.1	hypothetical protein OsJ_13133 [Oryza sativa Japonica Group]	1	207	21.5	5.66	2	207	21.5	5.66	0.500
XP_006654825.1	PREDICTED: GTP-binding nuclear protein Ran-2 [Oryza brachyantha]	1	221	25.0	7.12	2	221	25.0	7.12	0.500
XP_015617609.1	dehydrin Rab16B [Oryza sativa Japonica Group]	1	164	16.5	9.28	2	164	16.5	9.28	0.500
XP_015622758.1	copper transport protein ATX1 [Oryza sativa Japonica Group]	1	132	13.1	4.60	2	132	13.1	4.60	0.500
XP_015637493.1	oil body-associated protein 1A [Oryza sativa Japonica Group]	2	238	26.4	5.96	4	238	26.4	5.96	0.500
XP_015638298.1	gibberellin-regulated protein 2 [Oryza sativa Japonica Group]	1	92	9.7	8.44	2	92	9.7	8.44	0.500
XP_015639973.1	nuclear pore complex protein NUP50B [Oryza sativa Japonica Group]	1	472	49.7	4.77	2	472	49.7	4.77	0.500
XP_015644407.1	eukaryotic translation initiation factor 1A [Oryza sativa Japonica Group]	1	144	16.3	5.35	2	144	16.3	5.35	0.500
XP_015646663.1	alpha-amylase inhibitor 5 [Oryza sativa Japonica Group]	8	154	15.9	6.52	16	154	15.9	6.52	0.500
XP_015650252.1	NADP-dependent glyceraldehyde-3-phosphate dehydrogenase [Oryza sativa Japonica Group]	2	499	53.3	7.01	4	499	53.3	7.01	0.500
ACV65034.1	alpha-amylase inhibitor [Oryza sativa Indica Group]	18	179	19.2	8.19	37	179	19.2	8.19	0.486
EAY80813.1	hypothetical protein OsI_35993 [Oryza sativa Indica Group]	10	160	16.2	9.20	21	160	16.2	9.20	0.476
BAB71741.1	glyoxalase I [Oryza sativa Japonica Group]	29	291	32.5	5.67	65	291	32.5	5.67	0.446
AAB23101.1	bifunctional subtilisin/alpha-amylase inhibitor, RASI [Oryza sativa=rice, seeds, bran, Peptide, 176 aa] [Oryza sativa]	16	176	18.7	9.04	37	176	18.7	9.04	0.432
XP_015627797.1	hydroxyacylglutathione hydrolase cytoplasmic [Oryza sativa Japonica Group]	3	258	28.5	5.64	7	258	28.5	5.64	0.429
XP_015636388.1	ABA-inducible protein PHV A1 [Oryza sativa Japonica Group]	6	274	28.8	9.10	14	274	28.8	9.10	0.429
EAY88627.1	hypothetical protein OsI_10103 [Oryza sativa Indica Group]	12	276	27.1	5.05	30	276	27.1	5.05	0.400
EEE53911.1	hypothetical protein OsJ_00464 [Oryza sativa Japonica Group]	2	93	10.1	6.84	5	93	10.1	6.84	0.400
XP_015615102.1	non-specific lipid-transfer protein 1-like [Oryza sativa Japonica Group]	8	117	11.6	9.80	20	117	11.6	9.80	0.400
XP_015632174.1	actin-depolymerizing factor 4 [Oryza sativa Japonica Group]	2	139	15.9	6.04	5	139	15.9	6.04	0.400
BAA05537.1	WSI18 protein induced by water stress [Oryza sativa Japonica Group]	11	214	22.1	9.14	28	214	22.1	9.14	0.393
XP_015612430.1	uncharacterized protein LOC4324175 [Oryza sativa Japonica Group]	5	83	8.5	4.97	13	83	8.5	4.97	0.385
XP_015645223.1	alpha-amylase/trypsin inhibitor RA16 [Oryza sativa Japonica Group]	18	157	17.0	8.03	47	157	17.0	8.03	0.383
XP_015637572.1	late embryogenesis abundant protein 19 [Oryza sativa Japonica Group]	15	200	20.5	6.25	40	200	20.5	6.25	0.375
XP_015639340.1	aldose reductase [Oryza sativa Japonica Group]	6	318	35.6	6.80	16	318	35.6	6.80	0.375
CAA69949.1	lipid transfer protein [Oryza sativa]	10	116	11.4	9.13	28	116	11.4	9.13	0.357
ABC74439.1	differentiation embryo protein 31 [Oryza sativa Indica Group]	18	392	47.4	6.51	52	392	47.4	6.51	0.346
AAO37499.1	expressed protein [Oryza sativa Japonica Group]	1	158	16.5	4.46	3	158	16.5	4.46	0.333
ABA99939.2	Malate dehydrogenase, glyoxysomal precursor, putative, expressed [Oryza sativa Japonica Group]	1	320	33.6	7.62	3	320	33.6	7.62	0.333
CAI29542.1	eukaryotic translation initiation factor 2 alpha [Oryza sativa Indica Group]	1	339	38.2	5.40	3	339	38.2	5.40	0.333
EAY83219.1	hypothetical protein OsI_38428 [Oryza sativa Indica Group]	1	257	25.4	5.03	3	257	25.4	5.03	0.333
EAY84337.1	hypothetical protein OsI_05714 [Oryza sativa Indica Group]	1	131	13.8	10.24	3	131	13.8	10.24	0.333
EAZ11740.1	hypothetical protein OsJ_01605 [Oryza sativa Japonica Group]	1	467	49.8	6.28	3	467	49.8	6.28	0.333
EEC67044.1	hypothetical protein OsI_33786 [Oryza sativa Indica Group]	1	755	84.1	5.24	3	755	84.1	5.24	0.333
EEC67896.1	hypothetical protein OsI_35571 [Oryza sativa Indica Group]	1	347	36.6	8.51	3	347	36.6	8.51	0.333
XP_015611441.1	uncharacterized protein LOC4346534 [Oryza sativa Japonica Group]	1	95	10.3	4.81	3	95	10.3	4.81	0.333
XP_015620018.1	glycine-rich RNA-binding protein 2 [Oryza sativa Japonica Group]	2	162	16.1	6.74	6	162	16.1	6.74	0.333
XP_015611186.1	embryonic protein DC-8 [Oryza sativa Japonica Group]	5	241	24.5	6.40	16	241	24.5	6.40	0.313
BAS98064.1	Os06g0531200, partial [Oryza sativa Japonica Group]	2	289	30.9	5.78	7	289	30.9	5.78	0.286
ABG22475.1	Adenosylhomocysteinase, putative, expressed [Oryza sativa Japonica Group]	1	392	42.8	5.25	4	392	42.8	5.25	0.250
CAH67861.1	B0403H10-OSIGBa0105A11.13 [Oryza sativa]	1	110	12.3	8.50	4	110	12.3	8.50	0.250
EEC72589.1	hypothetical protein OsI_06045 [Oryza sativa Indica Group]	1	527	56.0	8.10	4	527	56.0	8.10	0.250
XP_015628800.1	defensin-like protein [Oryza sativa Japonica Group]	2	81	8.8	8.51	8	81	8.8	8.51	0.250
XP_008780868.1	class II metallothionein-like protein 1A [Phoenix dactylifera]	3	87	8.5	7.33	14	87	8.5	7.33	0.214

ABF95727.1	expressed protein [Oryza sativa Japonica Group]	1	279	30.8	6.86	5	279	30.8	6.86	0.200
BAD25501.1	hypothetical protein [Oryza sativa Japonica Group]	1	93	9.7	5.92	5	93	9.7	5.92	0.200
EAZ03220.1	hypothetical protein OsI_25368 [Oryza sativa Indica Group]	15	141	15.3	6.95	80	141	15.3	6.95	0.188
CAH67235.1	OSIGBa0140O07.3 [Oryza sativa]	1	215	23.2	5.54	6	215	23.2	5.54	0.167
XP_015625560.1	hsp70-Hsp90 organizing protein [Oryza sativa Japonica Group]	1	578	64.9	6.38	6	578	64.9	6.38	0.167
XP_015646664.1	seed allergenic protein RAG2-like [Oryza sativa Japonica Group]	32	160	17.3	8.34	241	160	17.3	8.34	0.133
XP_015643961.1	prolamin PPROL 17D-like [Oryza sativa Japonica Group]	1	149	16.7	7.90	8	149	16.7	7.90	0.125
ACA50505.1	seed allergenic protein RAG2 [Oryza sativa Japonica Group]	51	166	17.8	8.03	449	166	17.8	8.03	0.114
AAD10378.1	Bowman-Birk type trypsin inhibitor [Oryza sativa]	1	136	15.4	6.13	19	136	15.4	6.13	0.053
